# stageR: a general stage-wise method for controlling the gene-level false discovery rate in differential expression and differential transcript usage

**DOI:** 10.1186/s13059-017-1277-0

**Published:** 2017-08-07

**Authors:** Koen Van den Berge, Charlotte Soneson, Mark D. Robinson, Lieven Clement

**Affiliations:** 1 Department of Applied Mathematics, Computer Science and Statistics, Ghent University, Krijgslaan 281, S9, Ghent, 9000 Belgium; 2 Bioinformatics Institute Ghent, Ghent University, Ghent, 9000 Belgium; 3 Institute of Molecular Life Sciences, University of Zurich, Winterthurerstrasse 190, Zurich, 8057 Switzerland; 4 SIB Swiss Institute of Bioinformatics, University of Zurich, Zurich, 8057 Switzerland

**Keywords:** RNA-sequencing, Stage-wise testing, Differential transcript usage, Differential expression

## Abstract

RNA sequencing studies with complex designs and transcript-resolution analyses involve multiple hypotheses per gene; however, conventional approaches fail to control the false discovery rate (FDR) at gene level. We propose stageR, a two-stage testing paradigm that leverages the increased power of aggregated gene-level tests and allows post hoc assessment for significant genes. This method provides gene-level FDR control and boosts power for testing interaction effects. In transcript-level analysis, it provides a framework that performs powerful gene-level tests while maintaining biological interpretation at transcript-level resolution. The procedure is applicable whenever individual hypotheses can be aggregated, providing a unified framework for complex high-throughput experiments.

**Electronic supplementary material:**

The online version of this article (doi:10.1186/s13059-017-1277-0) contains supplementary material, which is available to authorized users.

## Background

High-throughput sequencing (HTS) technology has become the dominant platform for transcriptome profiling. It is agnostic of genomic annotation, has a broad dynamic range and allows data aggregation on different biological levels (basepair, exon, gene) [1–4]. Recent developments in read alignment provide fast transcript-level quantification [3, 5, 6], opening the way to assess differential transcript expression (DTE) and differential transcript usage (DTU), which for instance has been shown to be associated with Parkinson’s disease [7] and resistance to prostate cancer treatment [8]. In DTE, differential expression between conditions is assessed at the individual transcript level, while in DTU the relative expression of the isoforms of a gene are compared between conditions; i.e. a DTU analysis aims at discovering differences in the proportions of the expressed isoforms of a gene.

**Fig. 1:**
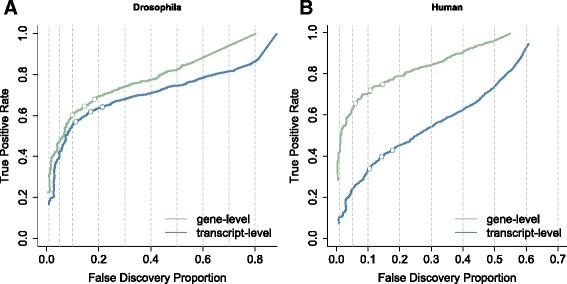
Performance curves for DTU analysis based on two simulation studies. The false discovery proportion (*FDP*, *x*-axis) is the fraction of false positive hypotheses over all rejected hypotheses. The true positive rate (*TPR*, *y*-axis) represents the fraction of false null hypotheses that have indeed been rejected. The three points on each curve represent working points on a nominal 1%, 5% and 10% FDR. The *left panel* **a** shows the results from a simulation performed in Soneson et al. (2016) [24] based on the *Drosophila melanogaster* transcriptome and clearly shows the increased sensitivity for tests that aggregate all transcript hypotheses on a gene level (*green curve*) in comparison to transcript-level tests (*blue curve*). The *right panel* **b** shows the results from a simulation based on the human transcriptome used in Soneson et al. (2016) [10]. Here, aggregated hypothesis tests show an even larger increase in sensitivity, possibly due to the higher complexity of the human transcriptome and thus a higher expected number of transcripts per gene for human

The dramatic sequencing cost reduction has also enabled researchers to set up studies with complex experimental designs involving many samples [9]. Analysis of DTU, DTE or traditional RNA-seq studies with complex designs typically involves multiple hypotheses of interest for each gene, e.g. for each transcript in a DTU and DTE context or for every treatment effect at each timepoint and the treatment-time interactions in time course differential gene expression (DGE) studies. The current consensus is to control the false discovery rate (FDR) on the hypothesis level, which we argue to be suboptimal with respect to statistical power and the downstream biological interpretation and validation that typically occur on a gene level. Soneson et al. [10] have shown that DTE analysis has higher performance when evidence on all individual transcripts is aggregated at the gene level due to the different null hypothesis and the larger amount of data that is available than for tests at the individual hypothesis level. This also occurs for DTU (see Fig. 1). Inference using *p* values of a DEXSeq [2] analysis on transcript counts also has a lower power than aggregating transcript-level *p* values to the gene level prior to FDR calculation. But, the latter does not provide identification of the specific transcripts that are differentially used; thus, higher sensitivity comes at the cost of a lower biological resolution.

**Fig. 2:**
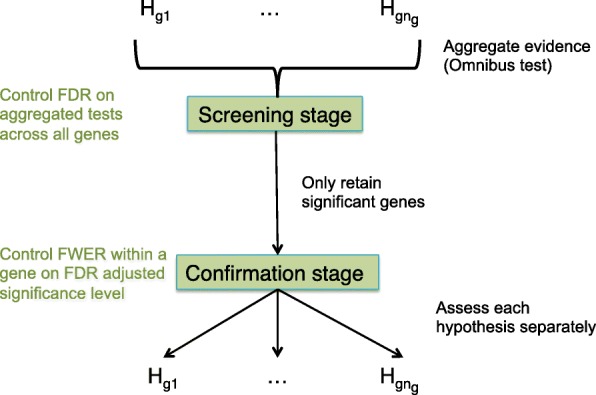
The stage-wise testing paradigm. *n*_*g*_ hypotheses are of interest for gene *g*. In the screening stage, evidence across the hypotheses is aggregated, and an omnibus test is performed that controls the FDR across all genes. Genes significant in the screening stage proceed to the confirmation stage, where every hypothesis is assessed separately, and the FWER within a gene is controlled at the adjusted significance level from the screening stage

In differential expression (DE) studies with complex designs, it is common practice to adopt multiple testing at the hypothesis level. This results in low power for discovering interaction effects since their standard error is typically much larger than for the main effects. Testing the treatment-time interaction effect in the cross-sectional time-series RNA-seq study from Hammer et al. (2010) [11] with limma-voom [12], for instance, returns no significant genes at a 5% FDR level, while more than 6000 genes are flagged when testing for treatment effects within a particular timepoint. Hence, the higher resolution on the hypothesis level comes at the expense of a low power for the interaction effect. In addition, FDR control on the hypothesis level does not guarantee FDR control on the gene level, because multiple hypotheses are assessed per gene, and the expected ratio of the number of genes with at least one false positive (false positive genes) to all positive genes in the union across hypotheses will be larger than the target FDR. For example, if three hypotheses are assessed with 5% false positives in the top-list for every contrast, then the aggregated top-lists will still contain 5% false positives. However, since the false positives in the different contrasts may be derived from different genes, the number of genes with false positives will increase with the number of hypotheses tested, while the total number of genes remains fixed. Thus, the gene-level FDR will be inflated if multiple hypotheses are of interest. This can lead to lower success rates of subsequent validation, since many genes without true treatment effects may be considered significant. In the RNA-seq literature, however, there is no consensus on how to combine the enhanced power of aggregation with an adequate resolution for the biological problem at hand. We argue that the multiple hypotheses at the gene level can be exploited in a two-stage testing procedure (Fig. 2) [13–15]. In the screening stage, genes with effects of interest are prioritised using an omnibus test, e.g. a global *F* test, a global likelihood ratio test or by aggregating *p* values. Assessing the aggregated null hypothesis has the advantages of (1) high sensitivity in a DTU/DTE context; (2) enriching for genes with significant interaction effects in complex DE studies, thereby boosting power; and (3) providing gene-level FDR control. In the confirmation stage, individual hypotheses are assessed for genes that pass the screening stage. Hence, it has the merit to combine the high power of aggregated hypothesis tests in stage I with the high resolution of individual hypothesis testing in stage II.

The suggested strategy positions itself in the larger framework of stage-wise testing procedures for high-throughput experiments. Lu et al. [16] previously proposed a two-stage strategy for microarrays based on mixed models, which is inapplicable to HTS data due to the violation of the distributional assumptions. Jiang and Doerge [13] proposed a generic two-stage DE analysis procedure where the first stage corresponds to testing a global null hypothesis, i.e. testing whether at least one hypothesis is false, after which post hoc tests are considered only for the significant genes. Their algorithm, however, relies on either distributional assumptions, providing a procedure for Gaussian distributed data, or computer intensive resampling-based techniques. For complex designs with a limited number of samples, however, the resampling-based techniques are not feasible. Under the condition that a gene with a true effect has a high probability to be rejected in the first stage, they also provide a method to guarantee an upper bound on the total FDR where the total FDR *α*≤*α*_1_+*α*_2_ with *α*_1_ and *α*_2_ the FDR levels of the first and the second stage, respectively. Due to the high level of noise in an RNA-seq context, this condition is not always fulfilled and the upper bound on the FDR is not guaranteed, but we would have to resort to the latter approach for a typical RNA-seq experiment with small sample sizes. Heller et al. [14] proposed a two-stage procedure in the context of gene set enrichment analysis (GSEA) for microarray data: in the screening stage, the global null hypothesis is tested for each gene set, and the procedure then tests for DE of individual genes within discovered gene sets. It was shown that their procedure controls the overall FDR (OFDR) [17], which provides error rate control on falsely discovered gene sets (Box 1) under independence, positive regression dependency and dependencies that are typically occurring in microarrays [14, 18, 19]. In this contribution, we port the ideas developed in Heller et al. [14] to the DTU, DTE and DE problem in HTS experiments with simple and complex designs by (1) replacing “a gene set” in their procedure by “a gene”, (2) aggregating evidence across all individual hypotheses per gene in the screening stage and (3) assessing each individual hypothesis on the discovered genes. In our context, the OFDR thus controls the FDR at the gene level, and we argue this to be the most appropriate error rate in complex high-throughput experiments due to its link with subsequent gene-level interpretation and biological validation. We further improve the power in the second stage of the Heller method by developing multiple testing procedures specifically tailored to the problem at hand. Similar to Meijer and Goeman [20, 21], our methods exploit the logical relations between the hypotheses that have to be assessed within each gene to reduce the multiple testing burden in the second stage. The procedure is powerful and easy to implement, and we will show that it provides an optimal middle ground between statistical power and resolution on the biological research questions for DGE, DTE and DTU analyses. It has been successfully applied in Moeys et al. [22], where a complex RNA-seq experiment assisted in the discovery of a pheromone-mediated sexual reproduction cycle of the diatom *Seminavis robusta*. The method has been implemented in an R package stageR available at https://github.com/statOmics/stageR.

## Box 1. OFDR and gene-level FDR

The overall FDR, or OFDR, was first defined in Benjamini and Heller (2008) [17]. It defines an FDR-like measure for error rate control if multiple hypotheses are assessed for every feature (e.g. gene or gene set). It controls the FDR at the feature level, i.e. over all hypotheses that are assessed for every feature. The OFDR is defined for stage-wise testing procedures that first discover interesting features using a screening hypothesis and assess the discovered features in a subsequent confirmation stage. Heller et al. (2009) [14] introduced the OFDR in the context of gene sets and define the OFDR as follows: “Let a discovered gene set be a gene set for which the screening hypothesis has been rejected, and let a falsely discovered gene set be a discovered gene set for which at least one null hypothesis (including possibly the screening hypothesis) was incorrectly rejected. The OFDR is the expected proportion of falsely discovered gene sets out of all discovered gene sets.” In this contribution we develop a stage-wise testing procedure for multi-factorial experiments or transcript-level analyses involving the assessment of multiple hypotheses per gene. By controlling the OFDR, we thus control the proportion of genes with at least one false null hypothesis over the total number of significant genes.

## Results

We evaluate the stage-wise testing procedure in DGE, DTE and DTU applications on both synthetic and real data. The results of the two-stage method are compared to the current consensus of a data analysis workflow in the specific applications (i.e. standard/conventional approach). First, we verify the gene-level FDR control and power for complex DGE experiments on simulated data upon which we confirm the simulation results on real data. We use the simulations to also compare to the stage-wise method from Jiang and Doerge [13], hereafter also referred to as the Jiang method. For DTE and DTU analyses, we show how the proposed stage-wise testing procedure maintains high sensitivity on the gene level while simultaneously providing a high resolution on the biological results. Additionally, we show that performance on the transcript level is at least as good as with a regular transcript-level analysis. By analysing a real prostate cancer dataset, we illustrate how the combination of gene and transcript-level results provides a rich resource for follow-up biological interpretation and validation.

### Differential gene expression

#### Simulation study

The simulation study is set up according to the Hammer study [11], a full factorial design with factors time (timepoint 1, timepoint 2) and treatment (control, spinal nerve ligation (SNL)) with two levels each. RNA-seq counts for 13,000 genes are simulated, 2000 genes have a constant fold change between treatment groups over time (main effect contrast), 2000 genes show DE in only one timepoint (1000 genes for every timepoint) and 1000 genes are differentially expressed in both timepoints with a different fold change between the timepoints (interaction effect). Thirty datasets are simulated with either five or three biological replicates in every treatment x time combination. The hypotheses of interest are DE at timepoint 1 and/or timepoint 2 (5000 genes) and testing for a change in DE between timepoints 1 and 2 (3000 genes with a real treatment × time interaction: 1000 genes with only DE in timepoint 1, 1000 genes with only DE in timepoint 2 and 1000 interaction genes with DE in both timepoints but with a differential fold change between them). A conventional approach assesses each of these hypotheses separately. Our two-stage approach, however, considers a test with an aggregated null hypothesis in the screening stage, i.e. that there is no effect of the treatment whatsoever. The individual hypotheses are only assessed in the confirmation stage for genes that passed the screening stage, i.e. for genes showing evidence for a treatment effect. The Jiang method assesses the same screening hypothesis in the first step, but it has the disadvantage that it must control the FDR on a lower level compared to our method, typically 80*%* of the total FDR as suggested by the authors [13], leading to a lower number of discovered genes. In the second step of the Jiang method, FDR is controlled on the union over all hypotheses on the remaining 20*%* level of the total FDR. We model the read counts by (generalised) linear models with a treatment effect, time effect and treatment x time interaction using the limma-voom (edgeR) [12, 23] framework, and a comparison is made in terms of FDR control, OFDR control and power.

**Fig. 3:**
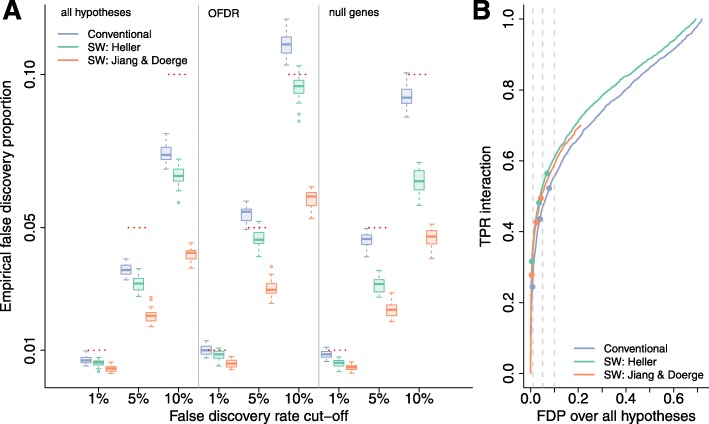
DGE simulation study results for the limma-voom analysis with five replicates in every treatment × time combination. **a** FDR and OFDR control for the conventional approach (*blue*), the stage-wise method proposed in this manuscript (*green*) and the stage-wise method from Jiang and Doerge [13] (*orange*). The false discovery proportion (*FDP*) is assessed in 30 simulations, which allows us to evaluate the FDR as the mean over all FDPs. The conventional method controls the FDR over all hypotheses but is too liberal on the OFDR, and the Jiang and Doerge method seems to be overly conservative in all scenarios. The stage-wise procedure we propose controls the FDR over all hypotheses; however, this is generally not guaranteed. As expected, it controls the OFDR on all significance levels. Compared to the conventional approach, the fraction of null genes (genes with no effect whatsoever) among the OFDR false positive list is lower for the stage-wise testing procedure proposed in this manuscript, which shows that it is advantageous in terms of efficient biological validation of the results. **b** False discovery proportion-true positive rate (*FDP-TPR*) performance curves for the treatment × time interaction effect based on the first simulation. The three points on the curves represent nominal FDR cut-offs at 1%, 5% and 10% and are filled if the empirical level is below its nominal level. The proposed stage-wise method boosts power for the interaction effect through the enrichment of interaction genes in the screening stage. The Jiang and Doerge method enriches for fewer genes as compared to the Heller method, because it has to split the FDR between its two stages. Furthermore, the Jiang and Doerge method is very conservative since it only allows control on the upper bound of the FDR across the hypotheses

Figure 3 shows the results for the limma-voom analysis of the simulated datasets with five biological replicates. All approaches control the FDR on the hypothesis level (i.e. across all returned hypotheses). This is expected for the conventional and Jiang methods, but is generally not guaranteed for our stage-wise approach since the latter is designed to control the OFDR. All methods have equivalent sensitivity for the individual hypotheses within each timepoint, but the Jiang method provides too conservative FDR control (Additional file 1: Figures S1 and S2). Since our proposed stage-wise testing procedure finds fewer genes (Additional file 1: Figures S3 and S4) with an equivalent (main effects) or higher (interaction) power combined with a lower OFDR, it enriches for genes with multiple effects. Moreover, the standard analysis leads to poor OFDR control, while the proposed stage-wise analysis controls the OFDR at its nominal level, indicating the superiority of a stage-wise testing approach to prioritise candidate genes for further analysis. In contrast, the Jiang method seems to be overly conservative. Biological validation and interpretation using tools like GSEA often occur at the gene level, which motivated us to compare the fraction of null genes (i.e. genes where all null hypotheses are true) in both candidate gene lists. When decomposing the OFDR false positive genes into (1) null genes and (2) genes with a treatment effect but where one of the true null hypotheses has been falsely rejected, we observe that the decrease in the fraction of false positives is more pronounced for the proposed stage-wise method than for the conventional analysis (Fig. 3, Additional file 1: Figure S5). Again, the Jiang method is too conservative. Thus, in addition to providing gene-level FDR control, the fraction of null genes among the false positive list is also lower for our method compared to the conventional approach, which will eventually limit resources wasted towards the validation of false positive genes in follow-up experiments.

In the introduction, we illustrated the low power of the conventional method for discovering interaction effects in RNA-seq experiments, and we observe a similar behaviour in the simulation study. Our two-stage method, however, enriches for genes with interaction effects by aggregating evidence across hypotheses in the screening step. In the simulation study with five replicates, this results in a power boost that can be observed for every fixed empirical false discovery proportion (FDP) (Figure 3 b, Additional file 1: Figure S6). Moreover, our stage-wise method, which is designed to control the OFDR, also controls the conventional FDR for the interaction effect (Additional file 1: Figure S7). The power boost at every FDP is even more pronounced in the 3 vs. 3 comparison (Additional file 1: Figure S6). The conventional FDR on the interaction effect, however, appears to be slightly too liberal in this comparison.

The results of the negative binomial edgeR analysis are qualitatively similar to the limma-voom analysis with the exception that FDR control can be rather liberal for edgeR, especially for small sample sizes (Additional file 1: Figures S8–S12). Hence, the proposed stage-wise analysis (1) returns a lower number of false positive genes and false positive null genes and (2) provides a higher power for interaction effects while (3) maintaining the same performance for the main effects.

#### Case study

We also re-analyse the Hammer dataset [11] with the two-stage method using limma-voom. We only compare the conventional and proposed stage-wise procedure, since the Jiang method was shown to have suboptimal performances in the simulation study. The results are very similar to those of the DGE simulation study, suggesting good quality of the simulated data: contrasts involving main effects have a similar number of significant genes between standard and stage-wise procedures, while the latter again finds many more significant genes when testing for the interaction effect (Table 1). While the conventional analysis did not find any genes when testing for the interaction effect, the stage-wise method retrieves 665 significant genes. The results of negative binomial count regression with edgeR are in line with the limma-voom analysis (Additional file 1: Table S1). Note, however, that edgeR does find 51 significant genes for the interaction test in a conventional analysis, which however may be a result of the higher power associated with edgeR’s rather liberal FDR control for experiments with small sample sizes (Additional file 1: Figure S12).

**Table 1:** Number of genes found in the Hammer dataset on a 5% FDR level in the limma-voom analysis

Procedure	t1	t2	Interaction	Stage I only	Unique genes
Standard	7125	6589	0	NA	8199
Stage-wise	6890	6574	665	85	7901

We considered 12,893 genes in the analysis

Eighty-five genes had only passed the screening stage, while none of them had a significant effect in the confirmation stage or by the standard analysis. Their expression profile reveals a moderate fold change with respect to the treatment that remains stable over time (Additional file 1: Figures S13 and S14), again indicating the higher sensitivity of the overall test in the screening stage. All genes, however, could be retrieved when testing for a contrast that quantifies the average fold change (i.e. average DE between SNL and control over time), confirming the biological relevance of the stage-wise testing approach, even for the genes without significant effects in the second stage. Note that incorporating the test for the average fold change does not alter the family-wise error rate (FWER) correction of the Shaffer method (see Methods) and can be adopted in the confirmation stage without compromising the power on the other contrasts of interest.

### Differential transcript usage and differential transcript expression

#### Simulation study

**Fig. 4:**
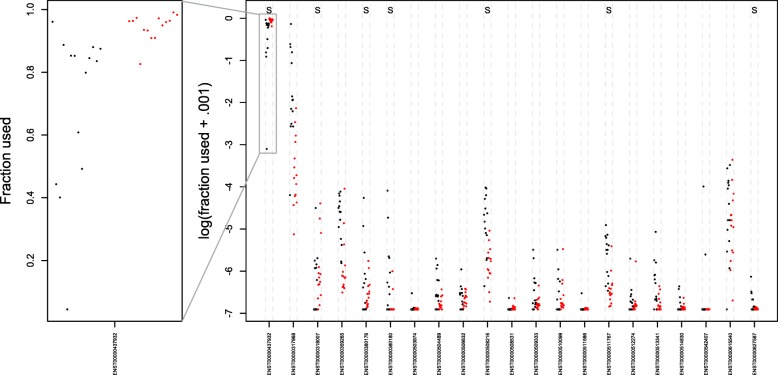
Expression pattern of the *PDLIM5* gene in the case study. The used fraction for every transcript is relative to the total expression of the genomic locus for a respective sample. *Black points* represent normal tissue, and *red points* represent tumoural tissue. The *left panel* (original scale) shows the dominant transcript that is additionally upregulated in tumoural tissue. The *right panel* shows the usage pattern on the log scale for all transcripts and shows that the upregulation of the dominant transcript is compensated for by a downregulation of multiple other transcripts. Significantly differentially used transcripts according to the stage-wise analysis are indicated with an S at the top of the plot

**Fig. 5:**
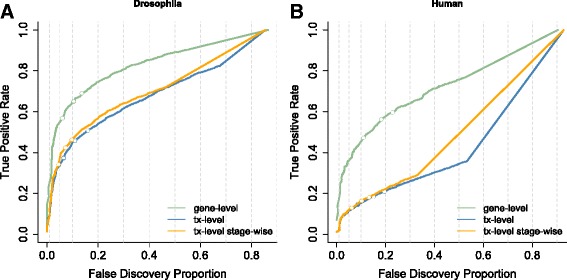
FDP-TPR performance curves for DTE analysis of the simulated data. *Blue curves* represent transcript-level tests, and *green curves* represent tests based on *p* values aggregated at the gene level. The *orange curve* represents the stage-wise transcript-level analysis. The three *open circles* on the curves represent working points for a target FDR of 1%, 5% and 10%. **a** Performance curve for the Drosophila simulation shows an inflated FDR for both the aggregated analysis on the gene level and a transcript-level analysis. The stage-wise transcript-level analysis has increased performance and additionally provides a better FDR control. **b** Performance curve for the human simulation shows even worse FDR control on all levels, which is in line with previous publications [24]. Similar to the Drosophila simulation, the transcript-level stage-wise analysis shows somewhat higher performance and provides a better FDR control

We adopt the *Drosophila melanogaster* and *Homo sapiens* simulation studies from Soneson et al. [24] for both DTE and DTU analyses. In the original simulation study DTU was simulated by flipping the proportions of the two most abundant transcripts, while in real data we often observe more than two significant transcripts per gene (see, e.g. Fig. 4). Hence, we have extended the simulation study to accommodate alternative splicing patterns across multiple transcripts per gene, and we also allow low-expressed genes to be simulated as differentially expressed or used. We compare the performance of the standard and stage-wise procedures both on the transcript and gene level by performing transcript-level tests or aggregating transcript-level *p* values, respectively. We did not consider analyses based on gene-level aggregated counts in the screening stage, because this approach would fail to find DTU for genes with constant output between conditions. The gene-level test is superior to the transcript-level test in terms of sensitivity for both DTE (Fig. 5) and DTU (Additional file 1: Figure S15), which motivates the stage-wise testing procedure. Furthermore, by leveraging the power in the gene-level test to the transcript-level analysis in the confirmation stage, the stage-wise analysis also has higher performance on the transcript level in a DTE analysis (Fig. 5) and is at least on par in a DTU analysis (Additional file 1: Figure S15) compared to a regular transcript-level approach. The stage-wise transcript-level tests do not only result in increased performance but additionally provide a better FDR control on the transcript level. Due to the better FDR control, the stage-wise analysis will not necessarily find more transcripts as compared to a transcript-level analysis, but the number of true positive transcripts for a fixed fraction of false positive transcripts in the rejected set should be at least identical or higher in the stage-wise analysis. We confirm the observation made in Soneson et al. [24] that FDR control deteriorates severely in human as compared to fruit fly and support their hypothesis that this is related to transcriptome complexity.

#### Case study

We analysed a prostate cancer dataset [25] from 14 Chinese tumour-normal matched samples for DTU and DTE. First, we use the case study dataset to evaluate the stage-wise testing procedure on real data. We only select the control samples and randomly assign group status resulting in a two-group 7 vs. 7 comparison. In order to simulate DTE/DTU, we randomly sample 1000 genes and a random number of transcripts within those genes (see “Methods” section for details). In the second group, we then swap expression values within the gene in the second condition. The performance results are qualitatively similar to the human simulation study, suggesting good quality of the simulated data (Additional file 1: Figure S16). Next, we evaluate DTU and DTE based on the original dataset. We use DEXSeq [2] for assessing DTU by providing transcript-level expression estimates instead of exon bin quantifications; hence, we fit transcript-level negative binomial models. In the screening stage, inference is based on transcript-level *p* values aggregated to the gene level, and transcripts of significant genes are confirmed using individual transcript-level tests. In the DTU analysis, we filter genes with only one transcript, which leaves 18,479 genes with a median of 6 transcripts per gene. On a 5% target OFDR level, the stage-wise testing analysis finds 4752 significant genes in the screening stage and confirms 6772 significant transcripts in the confirmation stage. Similarly, DTE is assessed using transcript-level negative binomial models implemented in edgeR [26], and gene-level tests are performed by aggregating transcript-level *p* values. For DTE, no filtering is required, so we consider 32,499 genes with a median of 2 transcripts per gene, although the analysis may benefit from independent low abundance filtering [27]. The stage-wise analysis finds 4842 significant genes in the screening stage and confirms 5236 transcripts in the second stage on a 5% target OFDR level.

The literature concerning alternative splicing has often focussed on isoform dominance, where it is hypothesised that many genes have dominant isoforms and a major mechanism of DTU would be a switch of dominance between two transcripts [28]. We find that two out of five genes (*CYP3A5* and *LPIN1*) with a gene-wise *q* value equal to zero have previously been associated with prostate cancer [29, 30], and indeed both genes seem to correspond to a switch in the dominant isoforms (Additional file 1: Figure S17). However, for both genes the association with prostate cancer was based on DE analysis, while we show that instead the underlying pattern of the previous results is due to DTU. We also observe that complex genes with many transcripts are much more likely to be flagged in a DTU analysis as compared to genes with a low number of isoforms (Additional file 1: Figure S18). Out of all significant genes with at least three transcripts, 10% have three or more transcripts confirmed as differentially used in the confirmation stage, providing accumulating evidence of more complex biological splicing patterns. According to the stage-wise testing method, two genes have seven or more differentially used transcripts on a 5% target OFDR level. The transcript usage pattern for one of them, the *PDLIM5* gene, corresponds to an upregulation for a dominant transcript that is compensated for by a downregulation of multiple others (Fig. 4, Additional file 1: Figure S19). Remarkably, a single nucleotide polymorphism (SNP) (rs17021918) in this gene was also found to be associated with prostate cancer in previous studies [31, 32], among which was a large-scale multi-stage genome-wide association study (GWAS) that provided robust associations across multiple populations [31]. When compared to a log-additive model, which is the most common model for association of SNPs with disease and assumes an additive effect of the log-odds on disease for each copy of the allele, the effect of rs17021918 exceptionally showed no difference in risk on prostate cancer between heterozygotes and homozygotes. Furthermore, the SNP lies in the intronic region of the *PDLIM5* gene and could contribute to alternative splicing patterns observed in prostate cancer cells as compared to normal cells instead of providing allele dosage effects on prostate cancer risk.

## Discussion

We adapted the two-stage procedure of Heller et al. [14] as a general inference paradigm to provide powerful statistical testing and FDR control for problems that allow hypotheses to be aggregated. We tailored it towards modern RNA-seq applications assessing multiple hypotheses per gene and showed that it is superior in terms of interpretation, sensitivity and specificity. The screening stage considers an omnibus test aggregating evidence across all hypotheses for every gene. This boosts the sensitivity for effects that have a relatively low power, e.g. interactions in studies with complex designs, and for picking up genes with differential transcript expression and transcript usage. Note that screening also results in a shift of the type of genes that are returned. The omnibus test might dilute the evidence of genes with a moderate effect for a single contrast (transcript) by aggregating it with the true null hypotheses for the remaining contrasts (transcripts). The loss of these genes, however, is compensated by the discovery of additional genes with moderate effect sizes for multiple contrasts of interest (transcripts) in studies with complex designs (DTE/DTU applications), e.g. the genes picked up in the Hammer study with a stable and moderate differential expression over time in rats with SNL compared to controls. Upon screening, individual effects/transcripts are further explored for the discovered genes. For RNA-seq applications, we also optimised the power of the confirmation stage by accounting for logical relations between the hypotheses that have to be assessed within each gene. In the confirmation stage, the proposed method is at least equivalent or slightly superior to the Jiang and Doerge method [13] in terms of sensitivity and specificity, while providing much better FDR control. In real applications, however, the Jiang method will always lead to suboptimal results for several reasons: (1) For RNA-seq experiments only an upper bound on the FDR can be guaranteed under the condition that genes with a true effect have a very high probability to pass the screening stage, which leads to very conservative results in our simulation studies. Note that this assumption, however, also might be violated, e.g. if many hypotheses are of interest due to dilution of the effects. (2) The screening stage is also bound to be less powerful than that of the Heller method, because the nominal FDR level has to be split over both stages. (3) It is unclear how the FDR is controlled for at the gene level.

We have focussed on time-series data in the application of the two-stage method on DGE analysis. However, the two-stage procedure is generic and can be applied to any design. For example, a DGE study that compares three drugs (e.g. a new drug, the current state of the art and a placebo) would require exactly the same data analysis paradigm as the Hammer dataset: three different hypotheses of interest (mean differential expression between the drugs) and, according to Shaffer’s modified sequentially rejective Bonferroni (MSRB) procedure, no correction is needed in stage II for FWER control. The extension to more complex designs is trivial when Holm’s method is used in stage II. However, a good understanding of the logical relations among the hypotheses is required for implementing Shaffer’s MSRB procedure so as to obtain maximal power.

The standard transcript-level and gene-level approaches for DTU/DTE analysis show an inflated FDR in all simulations. The stage-wise transcript-level analysis provides better FDR control, although it is often still inflated, especially for the human simulations. Indeed, the distribution of human transcript-level *p*-values is non-uniform for the higher *p* values (Additional file 1: Figure S20), suggesting invalid statistical inference, while a proper *p*-value distribution is observed for the Drosophila simulation (Additional file 1: Figure S20). Soneson et al. [24] suggested that the inflated FDR is related to transcriptome complexity and have shown that FDR control can become problematic for genes with many transcripts, which is partly due to the uncertainty associated with read attribution to similar transcripts. In addition, recent work has shown that transcript abundance estimates are infested with systematic errors as a result of a failure to model fragment GC content bias [33], leading to false positive transcript abundance results. Despite the computational advantage of light-weight algorithms like Salmon and kallisto [3, 6], many problems remain for correct estimation of transcript abundances, all of which may contribute to inflated FDR in transcript-level analyses. Even if the true transcript abundances could be obtained, the statistical inference engine of DEXSeq relies on large sample assumptions that are often not met in reality, and further method development is required to provide correct FDR control in a DTE/DTU context. Note, however, that our method is very general and can be easily adopted as new frameworks for DTE and DTU become available.

The DTU case study highlights the biological relevance of differential transcript usage in oncology research. It shows that prioritising genes in a first stage and subsequently confirming transcripts for the significant genes provides an elegant approach to DTU analysis, upon which biological interpretation of the results may follow. We confirm that a switch between dominant isoforms is a common pattern in DTU, but additionally provide evidence that the field may benefit from considering more complex splicing mechanisms as was shown for the *PDLIM5* gene. The stage-wise testing method provides an optimal data analysis strategy for discovering genes with dominant isoform switches as well as genes with more subtle changes in differential transcripts.

It is also important to stress that the stage-wise testing procedure has the merit to control the FDR at a gene level, which we claim to be beneficial over FDR control at a hypothesis level in experiments involving many hypotheses per gene. The gene is the natural level for downstream analysis, e.g. GSEAs and subsequent biological validation experiments. These might be compromised when using traditional hypothesis-level FDR control since the union of all genes found across hypotheses tends to be enriched for false positive genes for which all null hypotheses are true and that are not of interest to the biologist.

The Heller method controls the expected fraction of rejected genes with at least one false positive hypothesis and uses a FWER correction within a gene in the confirmation stage. Benjamini and Bogomolov [15] provide a general framework for such hierarchical stage-wise testing procedures. The method we propose could be considered as a special case of this general framework, where an FDR-based selection rule on the screening hypothesis is applied in the first stage and a FWER correction is applied in the second stage. However, the frameworks still differ in their FDR calculation. In the Benjamini and Bogomolov procedure, a false rejection of the screening hypothesis does not contribute to the FDR if there are no false rejections in the confirmation stage, while it does contribute in the Heller procedure. We propose the latter to be more relevant for the proposed applications since it incorporates false rejections across all hypotheses on the genes, and genes that were only rejected in the screening stage also provided meaningful biological results, as shown in the DGE case study. However, if many hypotheses are of interest over many genes (for example, a large-scale multi-trait GWAS [34]), then the framework from Benjamini and Bogomolov can be used to define a less stringent error measure, e.g. by using FDR control across the hypotheses within a gene in the confirmation stage.

In this contribution, we have deliberately chosen a stage-wise approach with nested hypothesis tests in the screening and confirmation stage. The method of Heller, however, does not imply the use of an omnibus test in the screening stage. In a DTE context, for instance, we also might opt to aggregate the transcript-level counts at a gene level instead of aggregating evidence over transcript-level hypotheses. We, however, feel that this will obscure the interpretation. In the latter approach, it is unclear how the screening step will enrich for the hypotheses of interest at a transcript level. For instance, genes with DTU and equal overall expression can exhibit a very clear DTE signal but are bound to fly under the radar when aggregating counts. The null hypothesis of the omnibus test in the screening stage has a natural interpretation that none of the effects of interest occur for a particular gene, vs. the alternative that at least one effect is present. Hence, the screening stage will enrich for genes with effects that will be further explored in the confirmation stage.

## Conclusions

We have introduced two-stage testing as a general paradigm for assessing high-throughput experiments involving multiple hypotheses that can be aggregated, which is implemented in the R package stageR (https://github.com/statOmics/stageR). We optimised the procedure towards RNA-seq applications: DTE, DTU and DGE analysis with simple and complex experimental designs. We have shown that the procedure controls the OFDR, which we argue to be the natural error rate in high-throughput studies: in our context the OFDR gets the interpretation of a gene-level FDR and shares a close link with the subsequent biological validation experiments and interpretation of the results, e.g. GSEAs. The omnibus test in the first stage boosts the power when testing for interaction effects in DE studies with complex designs, without compromising power on the remaining contrasts. For DTU and DTE analyses, the two-stage method gains from the high performance of FDR control upon aggregating transcript-level *p* values to the gene level. Specific transcripts can be identified in the subsequent confirmation stage for genes passing the screening stage. Hence, the two-stage procedure naturally unites the highest level of resolution on the biological problem with the superior power of aggregated hypothesis tests. In addition, the two-stage transcript-level analysis is on par (DTU) or has higher performances (DTE) than a conventional transcript-level analysis while providing better FDR control. We have used the two-stage testing procedure to prioritise interesting genes in a case study on prostate cancer, and we illustrated the potential of DTU analyses in the context of cancer research.

## Methods

### Two-stage testing procedure

The two-stage testing procedure that is proposed in this contribution was introduced by Heller et al. [14] for assessing GSEA. We adapt the procedure and formulate it more generally. Suppose we have a dataset that consists of *G* genes. For every gene *g*, we are interested in testing *n*_*g*_ null hypotheses $\phantom {\dot {i}\!}H_{1g},\ldots,H_{n_{g}g}$. In the screening stage the global null hypothesis $H_{g}^{S}$ is assessed, i.e. that all $\phantom {\dot {i}\!}H_{1g},\ldots,H_{n_{g}g}$ are true against the alternative hypothesis that at least one hypothesis *H*_*ig*_ is false. The confirmation stage consists of assessing all individual hypotheses $\phantom {\dot {i}\!}H_{1g},\ldots,H_{n_{g}g}$ for each gene that passed the screening stage. The procedure proceeds as follows:

1. Screening stage:
  Assess the screening hypothesis $H_{g}^{S}$ for all genes *g*=1,…,*G*.
  Let $p_{1}^{S},\ldots,p_{G}^{S}$ be the unadjusted *p* values from the screening stage test.
  Apply the Benjamini-Hochberg (BH) FDR procedure [35] to $p_{1}^{S},\ldots,p_{G}^{S}$ at FDR level *α*_*I*_. Let *R* be the number of rejected screening hypotheses.
2. Confirmation stage: For all *R* genes that pass the screening stage.
  Let *α*_*II*_=*R* *α*_*I*_/*G* be the BH-adjusted significance level from the first stage.
  Let $\phantom {\dot {i}\!}p_{1g}, \ldots, p_{n_{g}g}$ be the *p* values from $\phantom {\dot {i}\!}H_{1g},\ldots,H_{n_{g}g}$ for gene *g*.
  Adopt a multiple testing procedure to assess all *n*_*g*_ hypotheses while controlling the within-gene family-wise error rate (FWER) at the adjusted level *α*_*II*_.

Heller et al. [14] prove that the procedure controls the OFDR under independence between genes, an assumption that is required for the BH procedure. The BH procedure has been proven to be also valid under positive regression dependency [18] and it has additionally been shown to be valid under typical microarray dependencies between the genes [19, 36].

Note that any FWER correction procedure can be used in stage II. We, however, propose the use of the Shaffer MSRB method [37] when logical relationships amongst the hypotheses exist. The MSRB method is a modified Bonferroni procedure that accounts for the logical relationships among the hypotheses. Like the regular Bonferroni procedure, it is highly flexible and easily used in nonstandard situations of dependency [37]. In brief, the procedure works as follows. Suppose we have filtered the genes that pass the screening stage. Let $\phantom {\dot {i}\!}p_{(1)g},\ldots,p_{(n_{g})g}$ be the sorted unadjusted *p* values in the confirmation stage for gene *g* where $\phantom {\dot {i}\!}p_{(1)g} \le p_{(2)g} \le \ldots \le p_{(n_{g})g}$. The method works sequentially over the sorted *p* values: suppose that the first *j*−1 hypotheses have been rejected, we then compare *p*_(*j*)*g*_ to *α*_*II*_/*t*(*j*) where *t*(*j*) equals the maximum number of remaining hypotheses that still could be true given that the first *j*−1 hypotheses are false. *t*(*j*) is never greater than *n*_*g*_−*j*+1, and therefore the MSRB procedure uniformly outperforms the Holm [38] method.

In a standard setting, the first *p* value will be compared to *α*_*II*_/*t*(1)=*α*_*II*_/*n*_*g*_. However, within a two-stage procedure, we know that for every gene in the confirmation stage there is at least one effect, otherwise the screening hypothesis has been falsely rejected. Therefore, the MSRB procedure can be further modified such that the first *p* value can be compared to *α*_*II*_/(*n*_*g*_−1), hereby boosting power for the most significant test. Below, we show how *t*(*j*) might further reduce according to the specific context.

### Differential gene expression

#### Case study

The Hammer dataset [11] was downloaded from the ReCount [39, 40] project website (http://bowtie-bio.sourceforge.net/recount/). In this experiment, rats were subjected to a spinal nerve ligation (SNL), and transcriptome profiling occurred at 2 weeks and 2 months after treatment, for both the SNL group and a control group. Two biological replicates are used for every treatment × time combination. An independent filtering step [27] is performed prior to the analysis, after which we retain 12,893 genes with adequate expression (counts per million larger than 2) in at least two samples. Data was normalised using trimmed mean of *M* values (TMM) normalisation [23] to adjust for variations in sequencing depth and mRNA population. We analyse the data using a log-linear model implemented in limma-voom [12], and hypothesis testing is performed through moderated *t* tests and *F* tests. Additionally, we re-analyse the data using the negative binomial model implemented in edgeR [26] where hypothesis testing is performed through likelihood ratio tests. We assess (1) the treatment effect at the first timepoint, (2) the treatment effect at the second timepoint and (3) the treatment × time interaction using a contrast for the differential expression at the first and second timepoints and a difference in fold change between the two timepoints, respectively. For the standard analysis, every contrast has been assessed on a 5% target FDR level as was the screening hypothesis in the stage-wise analysis. When a gene correctly passes the screening hypothesis, at most one null hypothesis can still be true: there has to be DE at timepoint 1 or timepoint 2; if the DE only occurs on one timepoint, there also exists an interaction; if DE occurs at both timepoints, the *H*_0_ of no interaction can still be true. Of course, it is also possible that all null hypotheses are false. If a gene incorrectly passes the screening stage, it is a false positive by definition (see Box 1), no matter the results of the confirmation stage; i.e. further false positive rejections in the latter stage will not inflate the OFDR. Hence, *t*(3), *t*(2) and *t*(1)=1 in the Shaffer MSRB method for the Hammer experiment, and no additional FWER correction is required in the confirmation stage.

#### Simulation study

The simulation study is designed to mimic the Hammer dataset [11]. The raw count table was downloaded from the ReCount project [39, 40] website (http://bowtie-bio.sourceforge.net/recount/). We simulated realistic RNA-seq data based on the framework provided by [41] with some minor adjustments which allow us to link a gene’s characteristics over different timepoints, unlocking simulation of cross-sectional time-series DGE data. Gene-wise means *μ*_*g*_ and dispersions *ϕ*_*g*_ are estimated from the larger Pickrell dataset [42] for more efficient estimation and are jointly sampled for simulation, respecting the mean-variance relationship of RNA-seq data. We simulate 13,000 genes (equivalent to the number of genes analysed in the Hammer data) according to a negative binomial model for two timepoints and two conditions. We considered two sample sizes: simulated datasets with either five or three biological replicates for every treatment × time combination. We simulate 2000 genes with a constant fold change between control and treatment for both timepoints, 2000 genes with time-specific DE between treatment and control (1000 genes for every timepoint) and 1000 genes with a different fold change between timepoints (i.e. significant treatment × time interaction effect). All fold changes were set at 3 or 1/3, balanced in every contrast.

Similar to the case study, we use limma-voom [12] and edgeR [26] for DE analysis. As suggested by the authors [13], we implement the Jiang and Doerge stage-wise testing procedure by testing the screening hypothesis on a $\frac {4}{5}\alpha _{I}$ FDR level, where *α*_*I*_ refers to the screening stage FDR level for our proposed method. For the second stage, all confirmation hypothesis *p* values are aggregated and corrected for multiple testing on a $\frac {1}{5}\alpha _{I}$ FDR level. Following [14], we define a false positive gene as a gene where at least one of the null hypotheses (including the screening hypothesis) is falsely rejected and use this criterion to define the OFDR.

### Differential transcript usage and differential transcript expression

#### Simulation study

The simulation study is adapted from Soneson et al. [24]. In brief, RSEM [43] generates paired-end sequencing reads with a length of 101 bp based on parameters that are estimated from real RNA-seq data. The transcripts per million (TPM) expression levels and relative isoform abundances are estimated from real fastq files with RSEM: as in the original simulation study, we used sample SRR1501444 (http://www.ebi.ac.uk/ena/data/view/SRR1501444) for the Drosophila simulation and sample SRR493366 (http://www.ebi.ac.uk/ena/data/view/SRR493366) for the human simulation. Based on a mean-dispersion relationship derived from two real datasets (Pickrell [42] and Cheung [44] datasets, see [45]), every estimated expression level is matched with a corresponding negative binomial dispersion value for each gene. We scale the TPM values according to the desired library size to derive the gene-wise expected count and simulate counts from a negative binomial distribution. Two conditions were considered in the simulation study, and five samples were simulated in each condition. A Dirichlet distribution was used to simulate relative isoform abundances in each sample. We simulate 1000 genes with DTU. The genes with DTU were selected randomly from the subset of genes with expected gene count above 5 and at least two expressed isoforms. The number of differentially used transcripts within the gene is sampled ranging from a minimum of 2 up to a random number drawn from a binomial distribution with size equal to the number of transcripts and success probability 1/3. We introduce DTU by randomly flipping the proportions between the differentially used transcripts. In addition to the DTU genes we simulate 1000 DTE genes, where all transcripts from a gene are differentially expressed with fold changes drawn from a truncated exponential distribution. The simulated fastq files were mapped to the *Drosophila melanogaster* (*Homo sapiens*) transcriptome derived from the BDGP5.70 (GRCh37.71) primary genome assembly using kallisto [3].

#### Differential transcript usage

Prior to the analysis, we round the estimated isoform-level counts to the closest larger integer and discard genes with only one transcript and transcripts with no expression over all samples. The count matrix was then used as input to DEXSeq [2]. DEXSeq estimates size factors as in DESeq [46] for data normalisation. A transcript-wise negative binomial generalised linear model is fitted and changes in relative usage between the conditions are assessed by testing the transcript:condition interaction effect, comparing the expression ratio of the transcript over all other transcripts within a gene between conditions [24]. For the gene-level test, the transcript-level *p* values are aggregated to gene-level *q* values using the perGeneQValue function from DEXSeq [2], which amounts to controlling the FDR at level

$$ q^{*} = \frac{\sum_{g=1}^{G} 1-(1-\theta)^{n_{g}}}{R}, $$

with *G* the number of genes, *n*_*g*_ the number of transcripts for gene *g*, *θ* the significance threshold and *R* the number of rejections.

In the confirmation stage of the stage-wise analysis, we use the Shaffer MSRB method [37]. Genes in the confirmation stage have passed the screening stage; hence, at least one of the transcripts should be differentially used between conditions. Since one transcript is differentially used, the difference in usage must be compensated for by at least one other transcript. Hence, all genes passing the screening stage should at least have two, and possibly more, DTU transcripts. According to the Shaffer method, the two most significant transcripts can be tested at a significance level of *α*_*II*_/(*n*_*g*_−2), and from the third most significant transcript onwards the procedure reduces to the Holm method [38]. If a gene only consists of two transcripts, both are always called significant as soon as the gene passes the screening stage.

#### Differential transcript expression

Isoform-level estimated counts are rounded to the closest larger integer, and transcripts with no expression over all samples are discarded from the analysis. A negative binomial model is fit for every transcript using edgeR [23], and statistical inference is performed through likelihood ratio tests. For a transcript-level analysis the *p* values are adjusted using BH correction, while for a gene-level analysis they are aggregated to gene-level *q* values as described in the previous section. Similar to the DTU analysis, we account for the fact that genes in the confirmation stage must have at least one significant transcript; however, there is no further dependency between the hypotheses for DTE. Therefore, the Shaffer MSRB method only provides additional power for the most significant transcript, i.e. by testing it at *α*_*II*_/(*n*−1), and from the second transcript onwards it reduces to the Holm [38] method.

#### Case study

The unfiltered, unnormalised kallisto processed data was downloaded from The Lair project website (http://pachterlab.github.io/lair/) [47]. For the evaluation of our method using ground truth based on the case study dataset, we round the kallisto estimated transcript counts to the closest larger integer, only retain transcripts with 5 counts in at least 6 samples and remove genes with only one remaining transcript. We only select the control samples and randomly assign condition status resulting in a two-group 7 vs. 7 comparison. For a random sample of 1000 genes, the number of transcripts within a gene simulated to be differentially expressed/used is defined as the maximum of 2 and a random number from a binomial process with size equal to the number of isoforms and success probability 1/3. We simulate DTE and DTU by swapping the expression counts between the selected transcripts within the gene in the second condition and evaluate DTE/DTU as in the simulation study. For the analysis of the original dataset, we rounded the kallisto estimated transcript counts to the closest larger integer and removed genes with only one transcript for DTU analysis and transcripts with no expression over all samples for both DTU and DTE analysis. DTU was assessed using DEXSeq [2], and DTE analysis was performed using edgeR [23]. A patient block effect was added to account for the correlation between control and tumoural tissue within patients, and inference was performed as described in the simulation study. Both analyses were performed on a target 5% OFDR level. FWER correction in the confirmation stage of the stage-wise testing procedure was performed using an adapted Holm-Shaffer method [37], as described in the simulation study.

## Additional file

Additional file 1 Supplementary figures and tables. This file contains all supplementary figures and tables to the manuscript. (PDF 6 666 kb)

## Abbreviations

BH: Benjamini-Hochberg
DE: Differential expression
DGE: Differential gene expression
DTE: Differential transcript expression
DTU: Differential transcript usage
FDP: False discovery proportion
FDR: False discovery rate
FWER: Family-wise error rate
MSRB: Modified sequentially rejective Bonferroni
OFDR: Overall false discovery rate
SNL: Spinal nerve ligation
SNP: Single nucleotide polymorphism
TMM: Trimmed mean of M values
TPR: True positive rate
GWAS: genome-wide association study

## References

[1] Collado-Torres L, Nellore A, Frazee AC, Wilks C, Love MI, Langmead B, Irizarry RA, Leek JT, Jaffe AE. Flexible expressed region analysis for RNA-seq with derfinder. Nucleic Acids Res. 2016:852. doi:10.1093/nar/gkw852. 10.1093/nar/gkw852 PMC5314792 27694310

[2] Anders S, Reyes A, Huber W (2012). Detecting differential usage of exons from RNA-seq data. Genome Res.

[3] Bray NL, Pimentel H, Melsted P, Pachter L (2016). Near-optimal probabilistic RNA-seq quantification. Nat Biotechnol.

[4] Liao Y, Smyth GK, Shi W (2014). featureCounts: an efficient general purpose program for assigning sequence reads to genomic features. Bioinformatics (Oxford, England).

[5] Li B, Dewey CN (2011). RSEM: accurate transcript quantification from RNA-Seq data with or without a reference genome. BMC Bioinforma.

[6] Patro R, Duggal G, Love MI, Irizarry RA, Kingsford C (2017). Salmon provides fast and bias-aware quantification of transcript expression. Nature Methods.

[7] Rhinn H, Qiang L, Yamashita T, Rhee D, Zolin A, Vanti W, Abeliovich A, Dauer W, Przedborski S, Spillantini MG, Singleton AB, Polymeropoulos MH, Satake W, Simon-Sanchez J, Nalls MA, Wan OW, Chung KK, Burre J, Abeliovich A, Devi L, Raghavendran V, Prabhu BM, Avadhani NG, Anandatheerthavarada HK, Lesnick TG, Moran LB, Zhang Y, James M, Middleton FA, Davis RL, Zheng B, Hudson NJ, Reverter A, Dalrymple BP, Presson AP, D’Haeseleer P, Liang S, Somogyi R, Margolin AA, Reverter A, Hudson NJ, Nagaraj SH, Perez-Enciso M, Dalrymple BP, Myers AJ, Braak H, Webster JA, Kim J, Kuo YM, Mosharov EV, Park SS, Schulz EM, Lee D, Alberio T, Gomez-Santos C, Freedman ML, Lewis BP, Burge CB, Bartel DP, Vasudevan S, Tong Y, Steitz JA, Junn E, Sylvestre J, Margeot A, Jacq C, Dujardin G, Corral-Debrinski M, Corral-Debrinski M, Blugeon C, Jacq C, Russo A, Kamp F, Subramanian A, Goecks J, Nekrutenko A, Taylor J, Langmead B, Trapnell C, Pop M, Salzberg SL, Rhinn H, Qiang L, Yu W, Andreadis A, Luo M, Baas PW, Staropoli JF, Vonsattel JP, Amaya MPD, Keller CE, Gruber AR, Lorenz R, Bernhart SH, Neubock R, Hofacker IL (2012). Alternative *α*-synuclein transcript usage as a convergent mechanism in Parkinson’s disease pathology. Nat Commun.

[8] Antonarakis ES, Lu C, Wang H, Luber B, Nakazawa M, Roeser JC, Chen Y, Mohammad TA, Chen Y, Fedor HL, Lotan TL, Zheng Q, De Marzo AM, Isaacs JT, Isaacs WB, Nadal R, Paller CJ, Denmeade SR, Carducci MA, Eisenberger MA, Luo J (2014). AR-V7 and resistance to enzalutamide and abiraterone in prostate cancer. N Engl J Med.

[9] Goodwin S, McPherson JD, McCombie WR (2016). Coming of age: ten years of next-generation sequencing technologies. Nat Rev Genet.

[10] Soneson C, Love MI, Robinson MD (2016). Differential analyses for RNA-seq: transcript-level estimates improve gene-level inferences. F1000Research.

[11] Hammer P, Banck MS, Amberg R, Wang C, Petznick G, Luo S, Khrebtukova I, Schroth GP, Beyerlein P, Beutler AS (2010). mRNA-seq with agnostic splice site discovery for nervous system transcriptomics tested in chronic pain. Genome Res.

[12] Law CW, Chen Y, Shi W, Smyth GK (2014). voom: precision weights unlock linear model analysis tools for RNA-seq read counts. Genome Biol.

[13] Jiang H, Doerge RW (2006). A two-step multiple comparison procedure for a large number of tests and multiple treatments. Stat Appl Genet Mol Biol.

[14] Heller R, Manduchi E, Grant GR, Ewens WJ (2009). A flexible two-stage procedure for identifying gene sets that are differentially expressed. Bioinformatics (Oxford, England).

[15] Benjamini Y, Bogomolov M (2014). Selective inference on multiple families of hypotheses. J R Stat Soc Ser B (Stat Methodol).

[16] Lu Y, Zhu J, Liu P (2005). A two-step strategy for detecting differential gene expression in cDNA microarray data. Curr Genet.

[17] Benjamini Y, Heller R (2008). Screening for partial conjunction hypotheses. Biometrics.

[18] Benjamini Y, Yekutieli D (2001). The control of the false discovery rate in multiple testing under dependency. Ann Stat.

[19] Reiner A, Yekutieli D, Benjamini Y (2003). Identifying differentially expressed genes using false discovery rate controlling procedures. Bioinformatics (Oxford, England).

[20] Meijer RJ, Goeman JJ (2015). A multiple testing method for hypotheses structured in a directed acyclic graph. Biom J.

[21] Meijer RJ, Goeman JJ (2016). Multiple testing of gene sets from Gene Ontology: possibilities and pitfalls. Brief Bioinform.

[22] Moeys S, Frenkel J, Lembke C, Gillard JTF, Devos V, Van den Berge K, Bouillon B, Huysman MJJ, De Decker S, Scharf J, Bones A, Brembu T, Winge P, Sabbe K, Vuylsteke M, Clement L, De Veylder L, Pohnert G, Vyverman W (2016). A sex-inducing pheromone triggers cell cycle arrest and mate attraction in the diatom Seminavis robusta. Sci Rep.

[23] Robinson MD, Oshlack A (2010). A scaling normalization method for differential expression analysis of RNA-seq data. Genome Biol.

[24] Soneson C, Matthes KL, Nowicka M, Law CW, Robinson MD (2016). Isoform prefiltering improves performance of count-based methods for analysis of differential transcript usage. Genome Biol.

[25] Ren S, Peng Z, Mao JH, Yu Y, Yin C, Gao X, Cui Z, Zhang J, Yi K, Xu W, Chen C, Wang F, Guo X, Lu J, Yang J, Wei M, Tian Z, Guan Y, Tang L, Xu C, Wang L, Gao X, Tian W, Wang J, Yang H, Wang J, Sun Y (2012). RNA-seq analysis of prostate cancer in the Chinese population identifies recurrent gene fusions, cancer-associated long noncoding RNAs and aberrant alternative splicings. Cell Res.

[26] Robinson MD, McCarthy DJ, Smyth GK (2010). edgeR: a Bioconductor package for differential expression analysis of digital gene expression data. Bioinformatics (Oxford, England).

[27] Bourgon R, Gentleman R, Huber W (2010). Independent filtering increases detection power for high-throughput experiments. Proc Natl Acad Sci U S A.

[28] Gonzàlez-Porta M, Frankish A, Rung J, Harrow J, Brazma A (2013). Transcriptome analysis of human tissues and cell lines reveals one dominant transcript per gene. Genome Biol.

[29] Leskelä S, Honrado E, Montero-Conde C, Landa I, Cascón A, Letón R, Talavera P, Cózar JM, Concha A, Robledo M, Rodríguez-Antona C (2007). Cytochrome P450 3A5 is highly expressed in normal prostate cells but absent in prostate cancer. Endocr Relat Cancer.

[30] Brohee L, Demine S, Willems J, Arnould T, Colige AC, Deroanne CF (2015). Lipin-1 regulates cancer cell phenotype and is a potential target to potentiate rapamycin treatment. Oncotarget.

[31] Eeles RA, Kote-Jarai Z, Al Olama AA, Giles GG, Guy M, Severi G, Muir K, Hopper JL, Henderson BE, Haiman CA, Schleutker J, Hamdy FC, Neal DE, Donovan JL, Stanford JL, Ostrander EA, Ingles SA, John EM, Thibodeau SN, Schaid D, Park JY, Spurdle A, Clements J, Dickinson JL, Maier C, Vogel W, Dörk T, Rebbeck TR, Cooney KA, Cannon-Albright L, Chappuis PO, Hutter P, Zeegers M, Kaneva R, Zhang HW, Lu YJ, Foulkes WD, English DR, Leongamornlert DA, Tymrakiewicz M, Morrison J, Ardern-Jones AT, Hall AL, O’Brien LT, Wilkinson RA, Saunders EJ, Page EC, Sawyer EJ, Edwards SM, Dearnaley DP, Horwich A, Huddart RA, Khoo VS, Parker CC, Van As N, Woodhouse CJ, Thompson A, Christmas T, Ogden C, Cooper CS, Southey MC, Lophatananon A, Liu JF, Kolonel LN, Le Marchand L, Wahlfors T, Tammela TL, Auvinen A, Lewis SJ, Cox A, FitzGerald LM, Koopmeiners JS, Karyadi DM, Kwon EM, Stern MC, Corral R, Joshi AD, Shahabi A, McDonnell SK, Sellers TA, Pow-Sang J, Chambers S, Aitken J, Gardiner RAF, Batra J, Kedda MA, Lose F, Polanowski A, Patterson B, Serth J, Meyer A, Luedeke M, Stefflova K, Ray AM, Lange EM, Farnham J, Khan H, Slavov C, Mitkova A, Cao G, Easton DF (2009). Identification of seven new prostate cancer susceptibility loci through a genome-wide association study. Nat Genet.

[32] Sun J, Kader AK, Hsu FC, Kim ST, Zhu Y, Turner AR, Jin T, Zhang Z, Adolfsson J, Wiklund F, Zheng SL, Isaacs WB, Grönberg H, Xu J (2011). Inherited genetic markers discovered to date are able to identify a significant number of men at considerably elevated risk for prostate cancer. Prostate.

[33] Love MI, Hogenesch JB, Irizarry RA. Modeling of RNA-seq fragment sequence bias reduces systematic errors in transcript abundance estimation. Nat Biotechnol. 2016. doi:10.1038/nbt.3682. 10.1038/nbt.3682 PMC5143225 27669167

[34] Peterson CB, Bogomolov M, Benjamini Y, Sabatti C (2016). Many phenotypes without many false discoveries: error controlling strategies for multitrait association studies. Genet Epidemiol.

[35] Benjamini Y, Hochberg Y (1995). Controlling the false discovery rate: a practical and powerful approach to multiple testing. J R Stat Soc Series B (Methodological).

[36] Reiner-Benaim A (2007). FDR control by the BH procedure for two-sided correlated tests with implications to gene expression data analysis. Biom J Biom Z.

[37] Shaffer JP (1986). Modified sequentially rejective multiple test procedures. J Am Stat Assoc.

[38] Holm S (1979). A simple sequentially rejective multiple test procedure. Scand J Stat.

[39] Frazee AC, Langmead B, Leek JT (2011). ReCount: a multi-experiment resource of analysis-ready RNA-seq gene count datasets. BMC Bioinforma.

[40] Collado-Torres L, Nellore A, Kammers K, Ellis SE, Taub MA, Hansen KD, Jaffe AE, Langmead B, Leek JT (2017). Reproducible RNA-seq analysis using recount2. Nature Biotechnology.

[41] Zhou X, Lindsay H, Robinson MD (2014). Robustly detecting differential expression in RNA sequencing data using observation weights. Nucleic Acids Res.

[42] Pickrell JK, Marioni JC, Pai AA, Degner JF, Engelhardt BE, Nkadori E, Veyrieras JB, Stephens M, Gilad Y, Pritchard JK (2010). Understanding mechanisms underlying human gene expression variation with RNA sequencing. Nature.

[43] Li B, Dewey CN (2011). RSEM: accurate transcript quantification from RNA-Seq data with or without a reference genome. BMC Bioinforma.

[44] Cheung VG, Nayak RR, Wang IX, Elwyn S, Cousins SM, Morley M, Spielman RS (2010). Polymorphic cis- and trans-regulation of human gene expression. PLoS Biol.

[45] Soneson C, Delorenzi M (2013). A comparison of methods for differential expression analysis of RNA-seq data. BMC Bioinforma.

[46] Anders S, Huber W (2010). Differential expression analysis for sequence count data. Genome Biol.

[47] Pimentel H, Sturmfels P, Bray N, Melsted P, Pachter L (2016). The Lair: a resource for exploratory analysis of published RNA-Seq data. BMC Bioinforma.

