# Supplementary material for: stageR: a general stage-wise method for controlling the gene-level false discovery rate in differential expression and differential transcript usage
Source: Genome Biol. 2017 Aug 7;18:151. doi: 10.1186/s13059-017-1277-0 (PMC5547545; doi:10.1186/s13059-017-1277-0)
Supplement: Additional file 1 — Supplementary figures and tables. This file contains all supplementary figures and tables to the manuscript. (PDF 6 666 kb) [file 13059_2017_1277_MOESM1_ESM.pdf]

Additional file to "stageR: a general stage-wise method for controlling the gene-level false discovery rate in differential expression and differential transcript usage"

Koen Van den Berge[1,2], Charlotte Soneson[3,4], Mark D. Robinson[3,4], and Lieven Clement[1,2,*]

[1]Department of Applied Mathematics, Computer Science and Statistics, Ghent University, Ghent, Belgium
[2]Bioinformatics Institute Ghent, Ghent University
[3]Institute of Molecular Life Sciences, University of Zurich, Zurich, Switzerland
[4]SIB Swiss Institute of Bioinformatics, University of Zurich, Zurich, Switzerland
[*]Corresponding author; lieven.clement@ugent.be

S-1.1 Differential Gene Expression Simulations

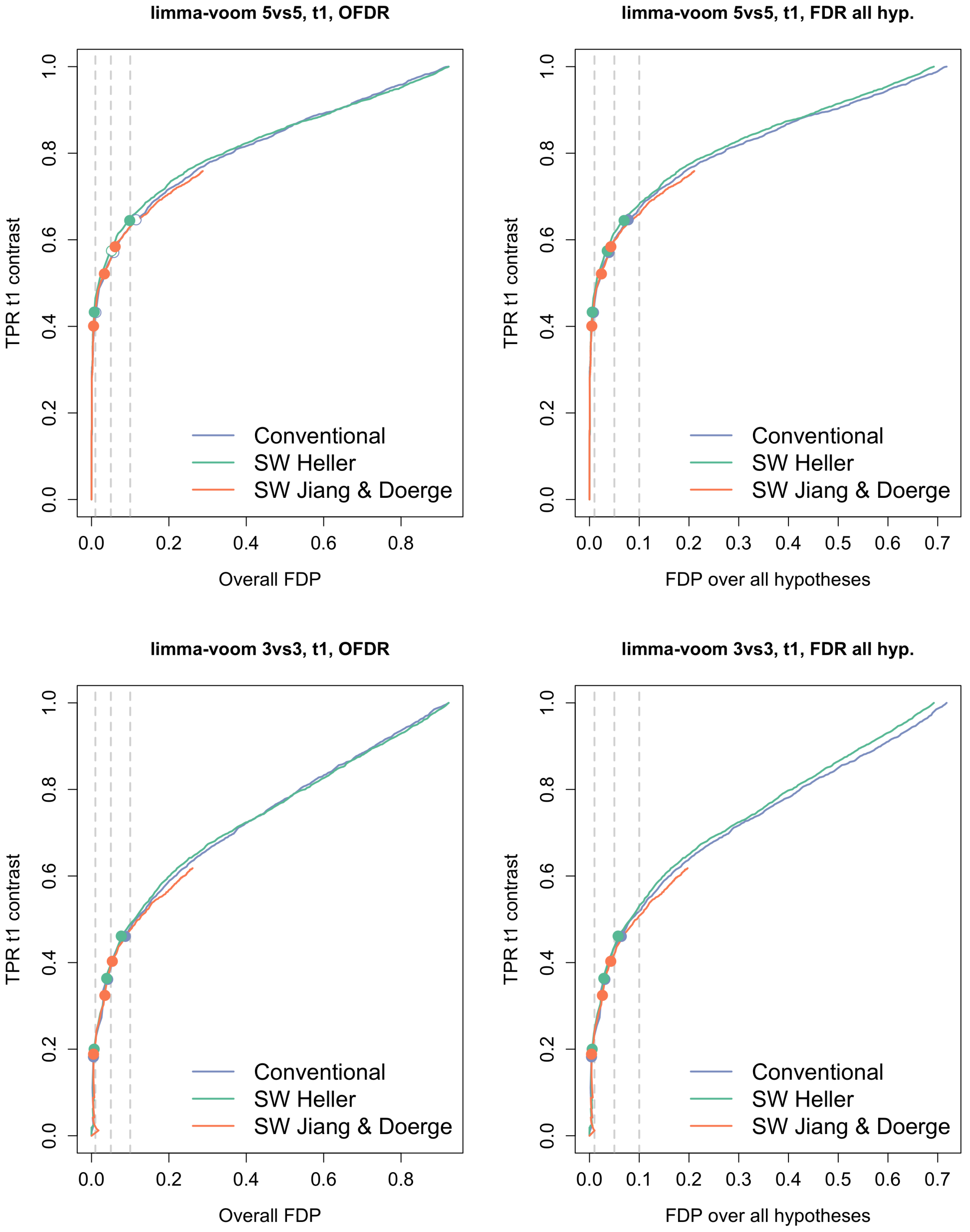

Figure S1: False discovery proportion - True positive rate (FDP-TPR) performance curves for the contrast at the first timepoint with the limma-voom analysis. Top row: comparisons with five samples in every condition, bottom row: comparisons with three samples in every condition, left panels: performance of the overall FDR control and right panels: performance of FDR control across all hypotheses. The three points on the curves represent nominal FDR cut-offs at 1%, 5% and 10% and filled symbols indicate that the empirical level is below its nominal level. A comparable sensitivity is observed across the three methods, however the Jiang method has conservative FDR control as suggested by the FDR working points.

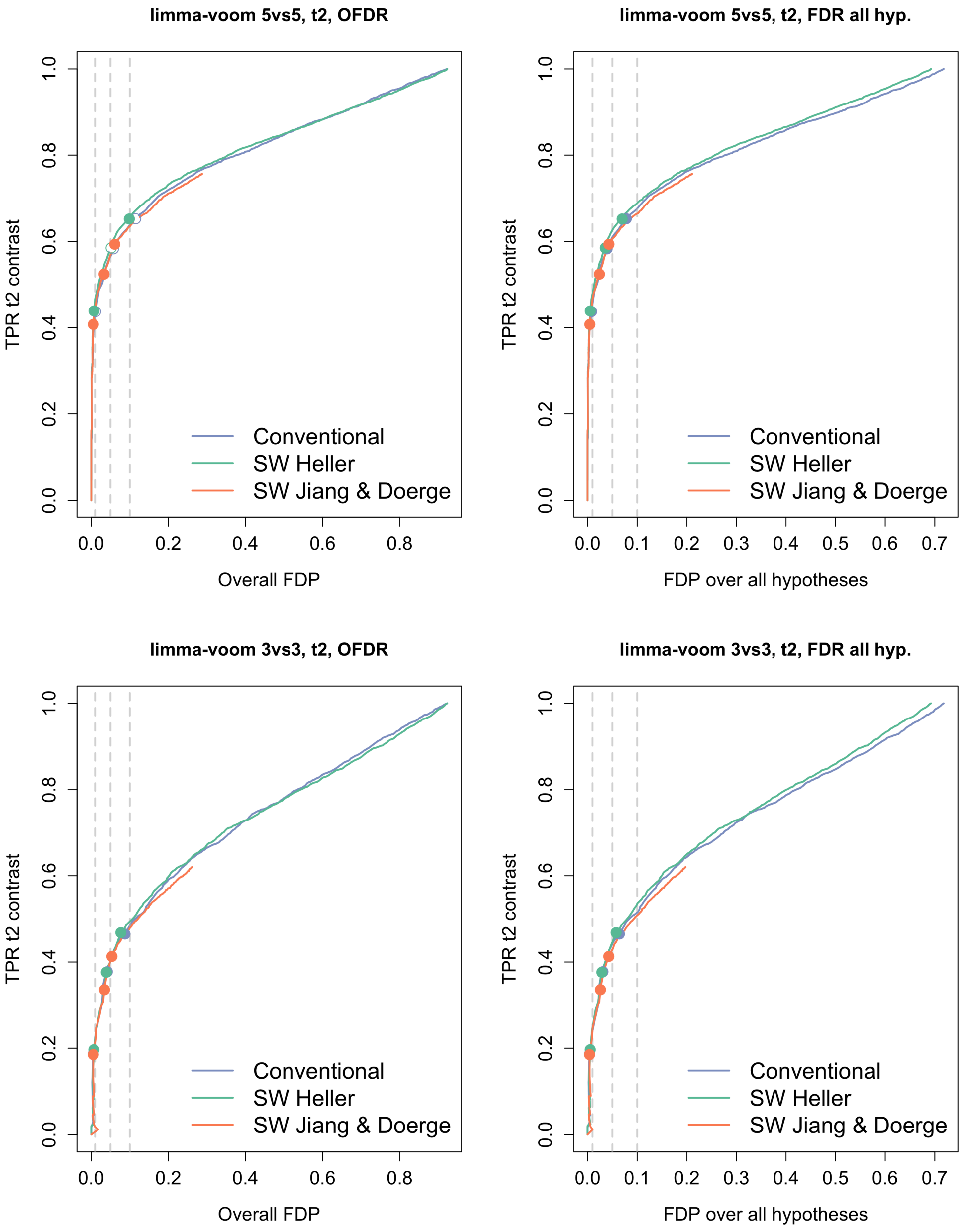

Figure S2: False discovery proportion - True positive rate (FDP-TPR) performance curves for the contrast at the second timepoint with the limma-voom analysis. Top row: comparisons with five samples in every condition, bottom row: comparisons with three samples in every condition, left panels: performance of the overall FDR control and right panels: performance of FDR control across all hypotheses. The three points on the curves represent nominal FDR cut-offs at 1%, 5% and 10% and filled symbols indicate that the empirical level is below its nominal level. A comparable sensitivity is observed across the three methods, however the Jiang method has conservative FDR control as suggested by the FDR working points.

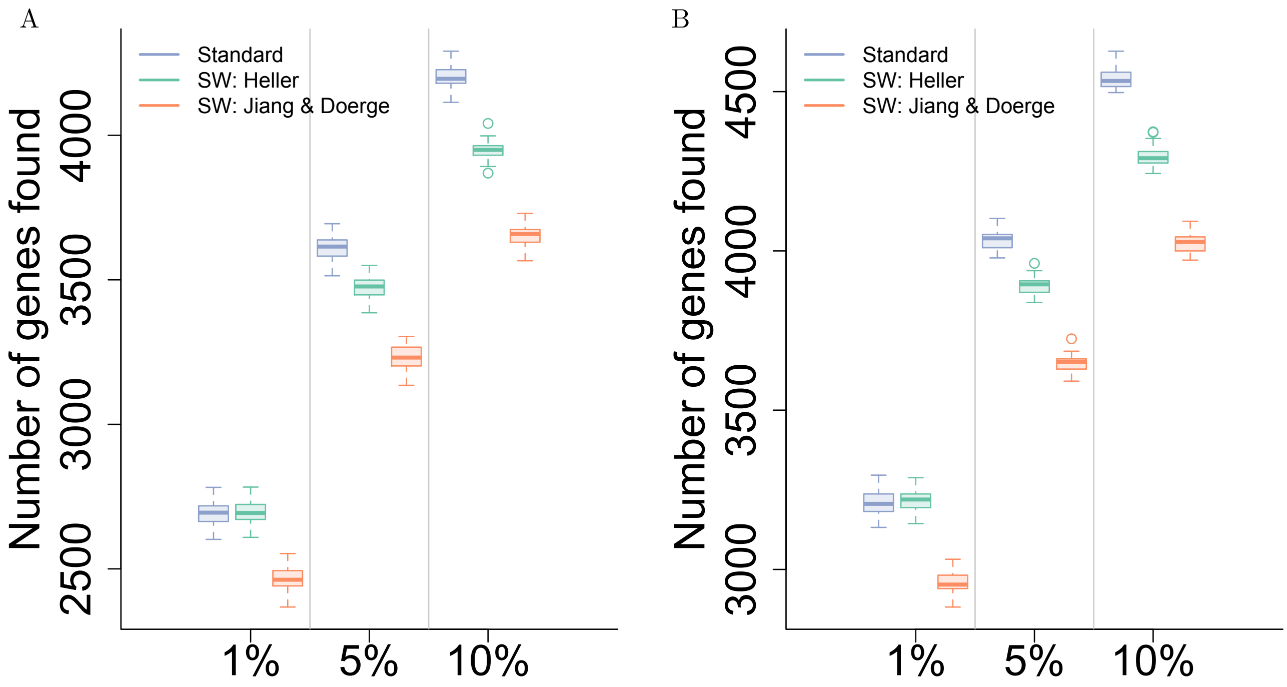

Figure S3: The total number of genes found in the five replicate simulation for a (A) limma-voom and (B) edgeR analysis is larger in a conventional analysis compared to the proposed stage-wise analysis on target FDR levels of 5% and 10%, while it is equivalent in a stage-wise analysis on a target FDR level of 1%. The Jiang procedure consistently reports a lower number of genes.

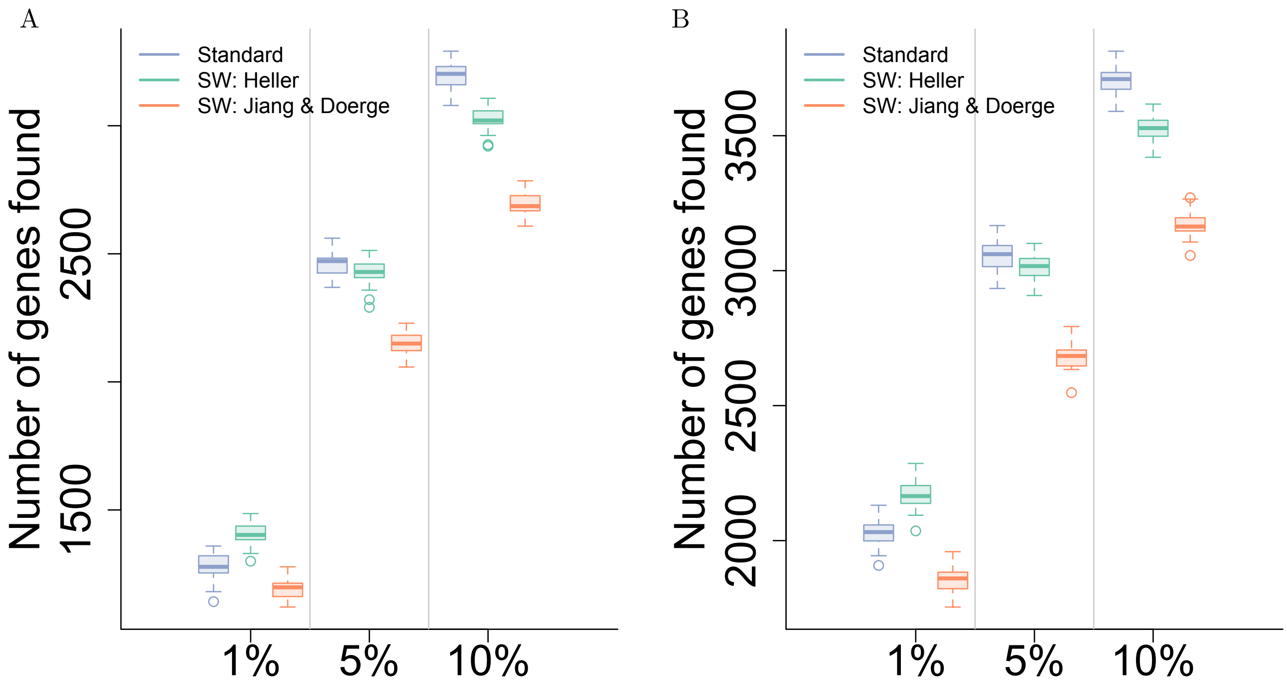

Figure S4: The total number of genes found in the three replicate simulation for a (A) limma-voom and (B) edgeR analysis is larger in a conventional analysis compared to the proposed stage-wise analysis on target FDR levels of 5% and 10%, while it is larger in a stage-wise analysis on a target FDR level of 1%. The Jiang procedure consistently reports a lower number of genes.

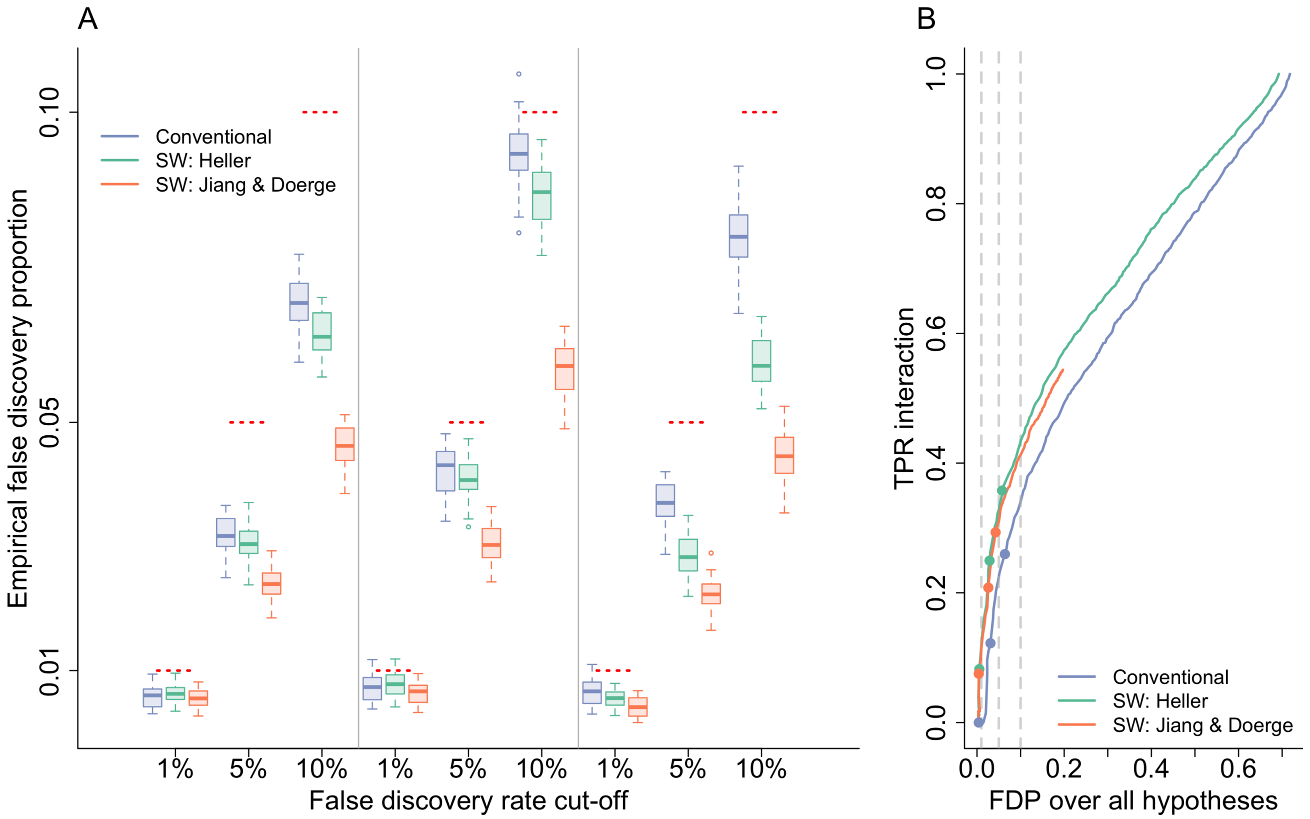

Figure S5: DGE simulation study results for the limma-voom analysis with three replicates in every treatment × time combination. (A) FDR and OFDR control for the conventional approach (blue), the stage-wise method proposed in this manuscript (green) and the stage-wise method from Jiang & Doerge [1] (orange). The false discovery proportion (FDP) is assessed in 30 simulations which allows us to evaluate the FDR as the mean over all FDPs. Both the standard and the proposed stage-wise approach control the FDR over all hypotheses, which is expected for the conventional method but not for the stage-wise analysis. The Jiang procedure seems to be overly conservative in all scenarios. OFDR is controlled for both the standard and our stage-wise analysis, although OFDR control is only guaranteed in the stage-wise analysis. Compared to the conventional approach, the fraction of null genes among the OFDR false positive list is lower for the proposed the stage-wise testing procedure, which shows that it is advantageous in terms of efficient biological validation of the results. (B) False discovery proportion - True positive rate (FDP-TPR) performance curves for the treatment × time interaction effect based on the first simulation. The three points on the curves represent nominal FDR cut-offs at 1%, 5% and 10% and are filled if the empirical level is below its nominal level. The stage-wise methods boost power for the interaction effect through the enrichment of interaction genes in the screening stage, however more genes are enriched in the Heller method. Furthermore, the Jiang & Doerge [1] method is very conservative since it only allows control on the upper-bound of the FDR across the hypotheses.

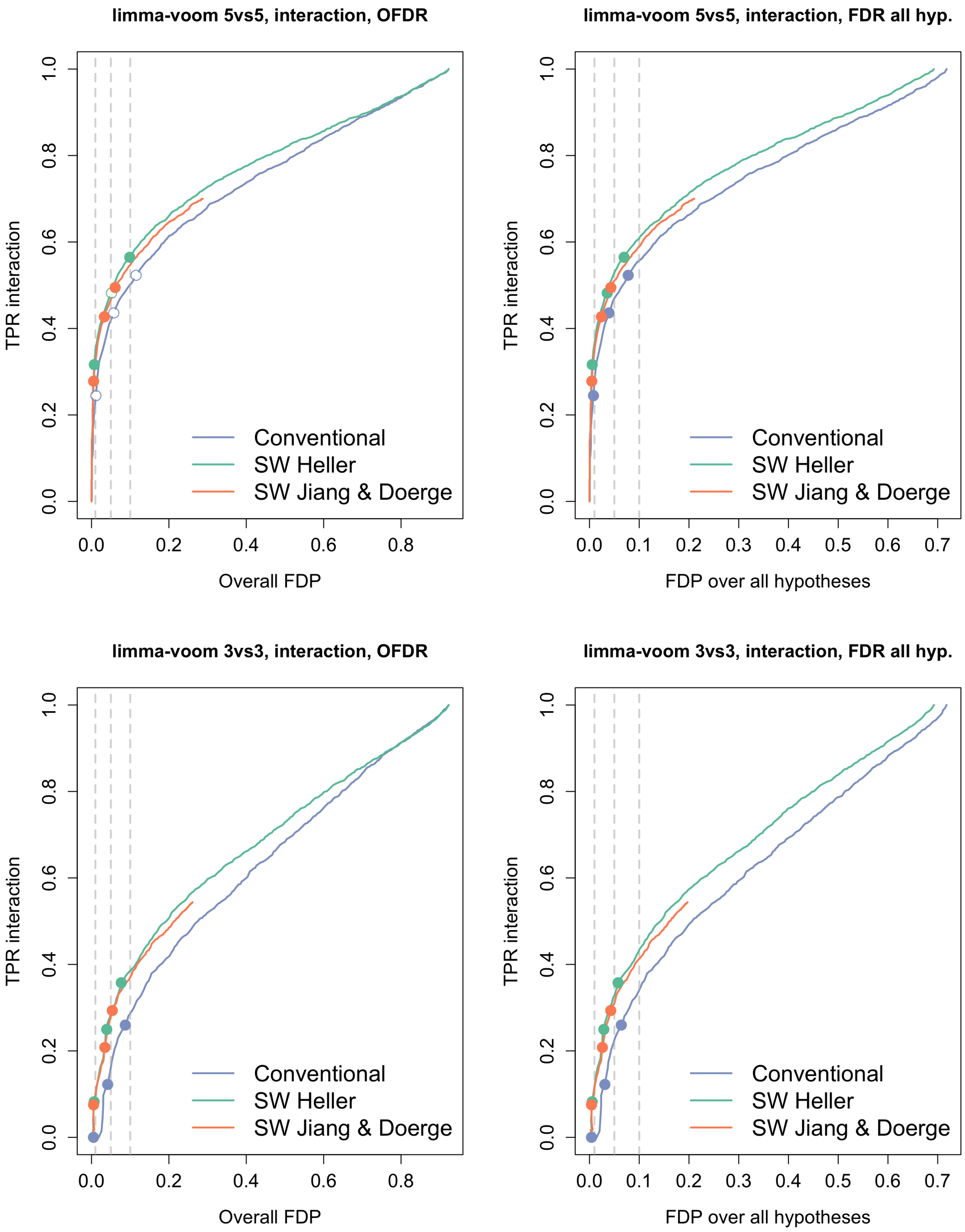

Figure S6: False discovery proportion - True positive rate (FDP-TPR) performance curves for the interaction effect with the limma-voom analysis. Top row: comparisons with five samples in every condition, bottom row: comparisons with three samples in every condition, left panels: performance of the overall FDR control and right panels: performance of FDR control across all hypotheses. The three points on the curves represent nominal FDR cut-offs at 1%, 5% and 10% and filled symbols indicate that the empirical level is below its nominal level. A superior sensitivity is observed for the Heller and the Jiang stage-wise methods as compared to the conventional method. The Jiang method, however, provides a very conservative FDR control.

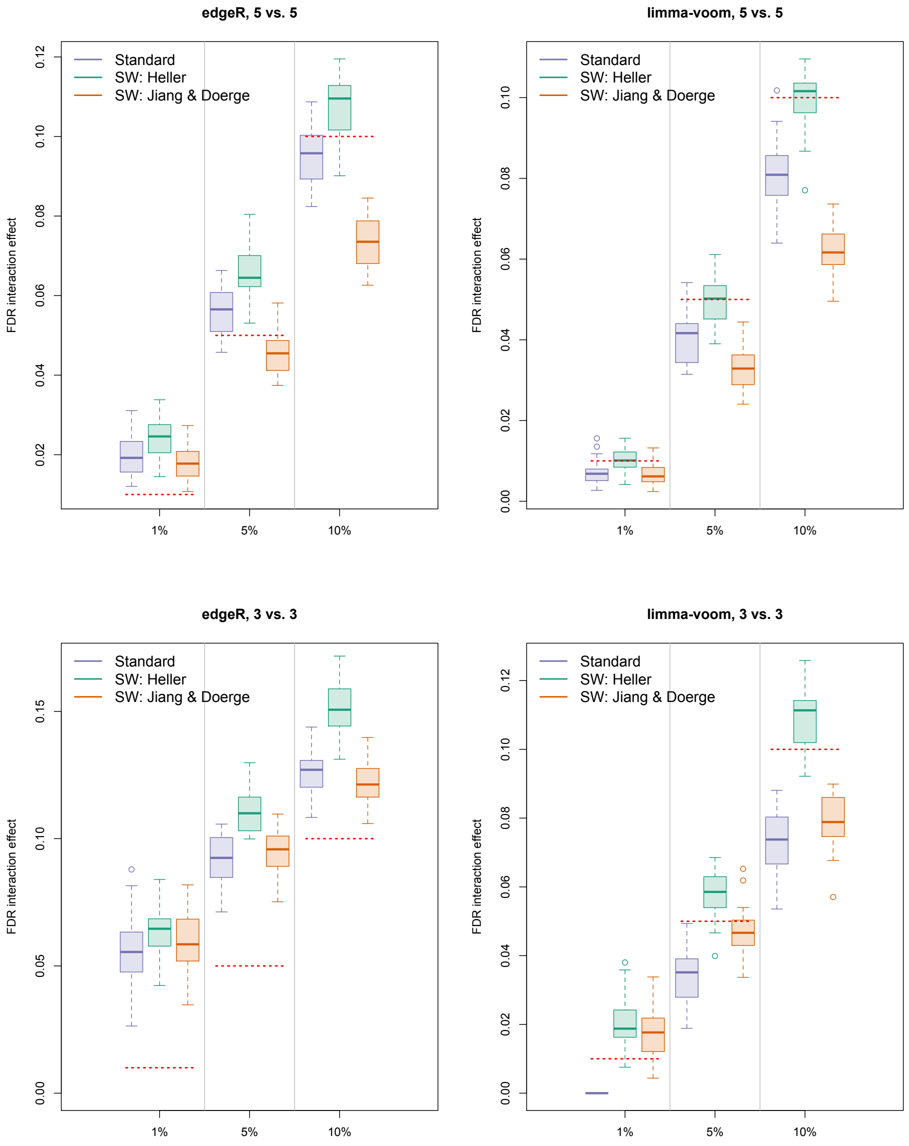

Figure S7: Evaluation of the false discovery rate on the interaction effect. Top row: comparisons with five samples in every condition, bottom row: comparisons with three samples in every condition, left panels: edgeR analysis and right panels: limma-voom analysis. Although that the Heller method is designed to control the overall FDR, it also controls the conventional FDR for the interaction in the 5 vs. 5 limma-voom simulations and it is somewhat more liberal for lower sample sizes. The FDR control is in general too liberal for the edgeR analysis.

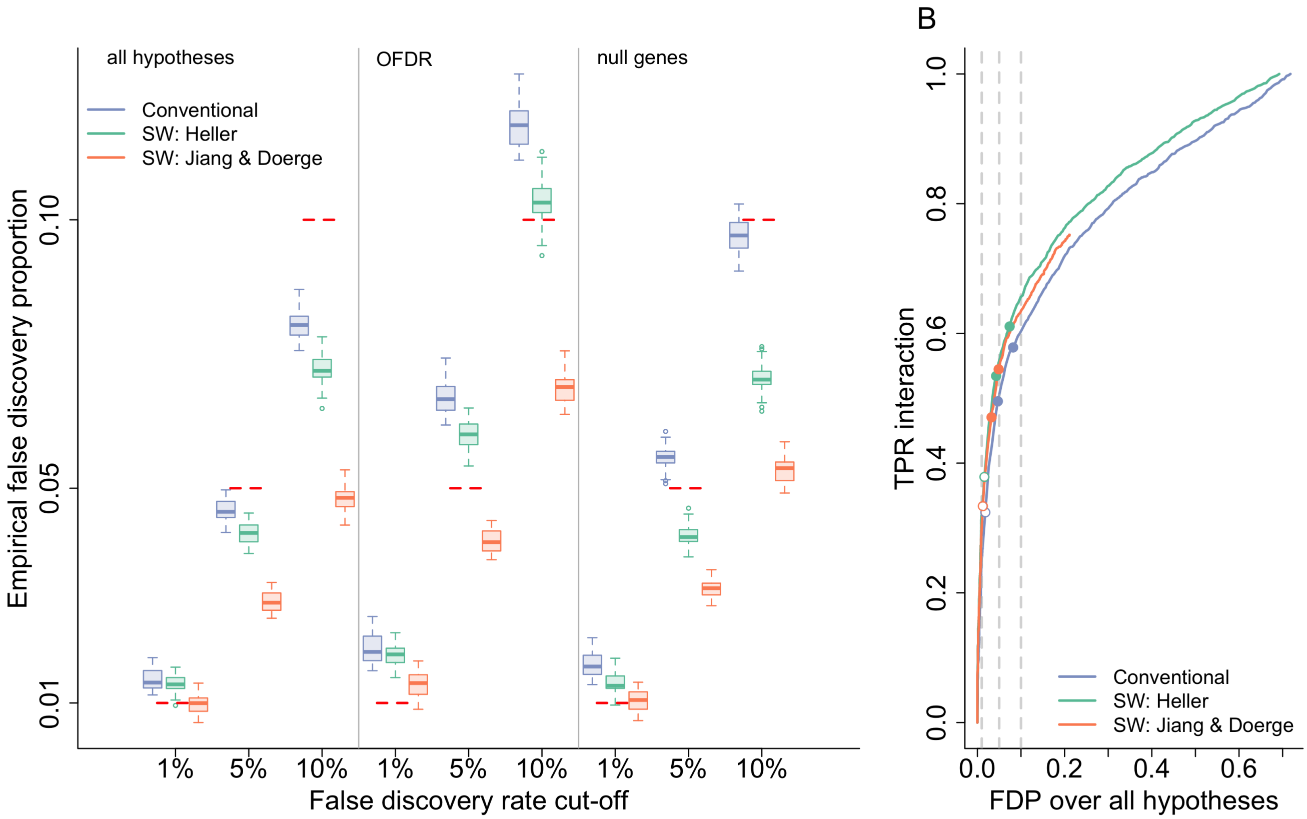

Figure S8: DGE simulation study results for the edgeR analysis with five replicates in every treatment $\times$ time combination. (A) FDR and OFDR control for the conventional approach (blue), the stage-wise method proposed in this manuscript (green) and the stage-wise method from Jiang & Doerge [1] (orange). The false discovery proportion (FDP) is assessed in 30 simulations which allows us to evaluate the FDR as the mean over all FDPs. Both the standard and the proposed stage-wise approach control the FDR over all hypotheses, which is expected for the conventional method but not for the stage-wise analysis. The Jiang procedure seems to be overly conservative in all scenarios. OFDR control is too liberal for edgeR in both the standard and stage-wise analysis, although OFDR control is better in the stage-wise analysis. Compared to the conventional appraoch, the fraction of null genes among the OFDR false positive list is lower for our proposed stage-wise testing procedure, which shows that it is advantageous in terms of efficient biological validation of the results. (B) False discovery proportion - True positive rate (FDP-TPR) performance curves for the treatment $\times$ time interaction effect based on the first simulation. The three points on the curves represent nominal FDR cut-offs at 1%, 5% and 10% and are filled if the empirical level is below its nominal level. The stage-wise methods boost power for the interaction effect through the enrichment of interaction genes in the screening stage, however more genes are enriched in the Heller method. Furthermore, the Jiang & Doerge [1] method is very conservative since it only allows control on the upper-bound of the FDR across the hypotheses.

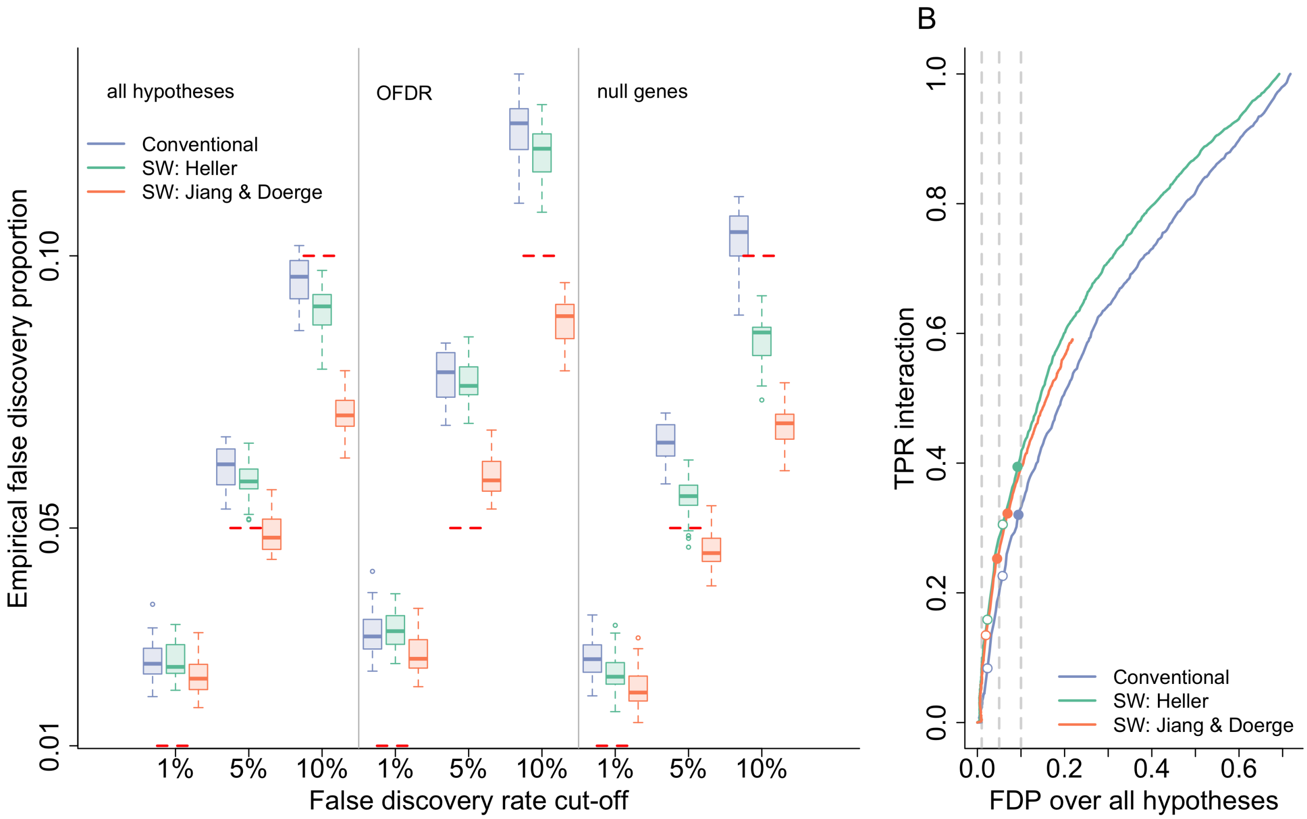

Figure S9: DGE simulation study results for the edgeR analysis with three replicates in every treatment × time combination. (A) FDR and OFDR control for the conventional approach (blue), the stage-wise method proposed in this manuscript (green) and the stage-wise method from Jiang & Doerge [1] (orange). The false discovery proportion (FDP) is assessed in 30 simulations which allows us to evaluate the FDR as the mean over all FDPs. Both the standard and the proposed stage-wise approach control the FDR over all hypotheses, which is expected for the conventional method but not for the stage-wise analysis. The Jiang procedure seems to be overly conservative in all scenarios. OFDR control is too liberal for edgeR in both the standard and stage-wise analysis, although OFDR control is better in the stage-wise analysis. Compared to the conventional appraoch, the fraction of null genes among the OFDR false positive list is lower for our proposed stage-wise testing procedure, which shows that it is advantageous in terms of efficient biological validation of the results. (B) False discovery proportion - True positive rate (FDP-TPR) performance curves for the treatment × time interaction effect based on the first simulation. The three points on the curves represent nominal FDR cut-offs at 1%, 5% and 10% and are filled if the empirical level is below its nominal level. The stage-wise methods boost power for the interaction effect through the enrichment of interaction genes in the screening stage, however more genes are enriched in the Heller method. Furthermore, the Jiang & Doerge [1] method is very conservative since it only allows control on the upper-bound of the FDR across the hypotheses.

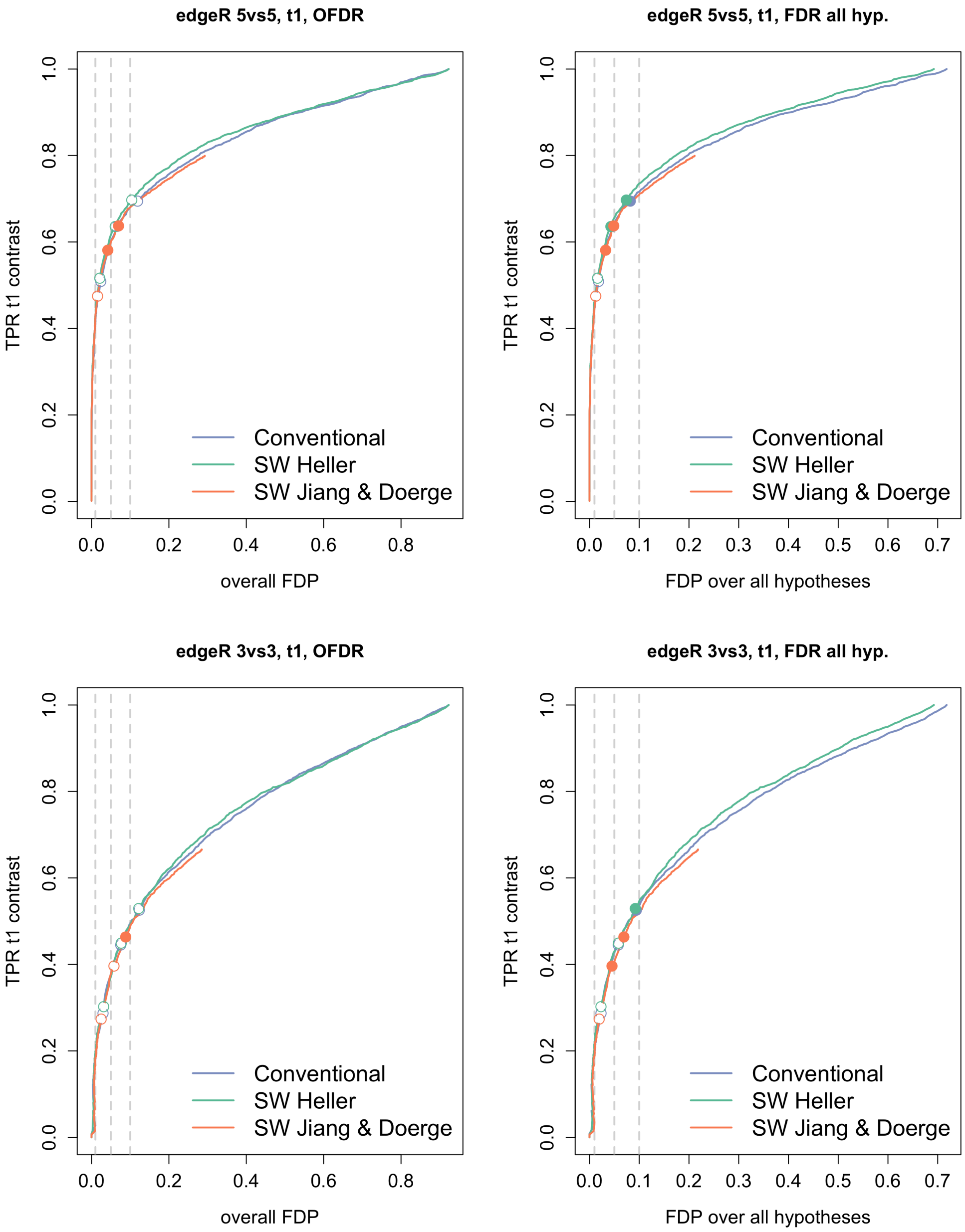

Figure S10: False discovery proportion - True positive rate (FDP-TPR) performance curves for the contrast at the first timepoint with the edgeR analysis. Top row: comparisons with five samples, bottom row: comparisons with three samples in every condition, left panels: performance of the overall FDR control and right panels: performance of FDR control across all hypotheses. The three points on the curves represent nominal FDR cut-offs at 1%, 5% and 10% and are filled if the empirical level is below its nominal level. A comparable sensitivity is observed across the three methods, however the Jiang method has conservative FDR control as suggested by the FDR working points.

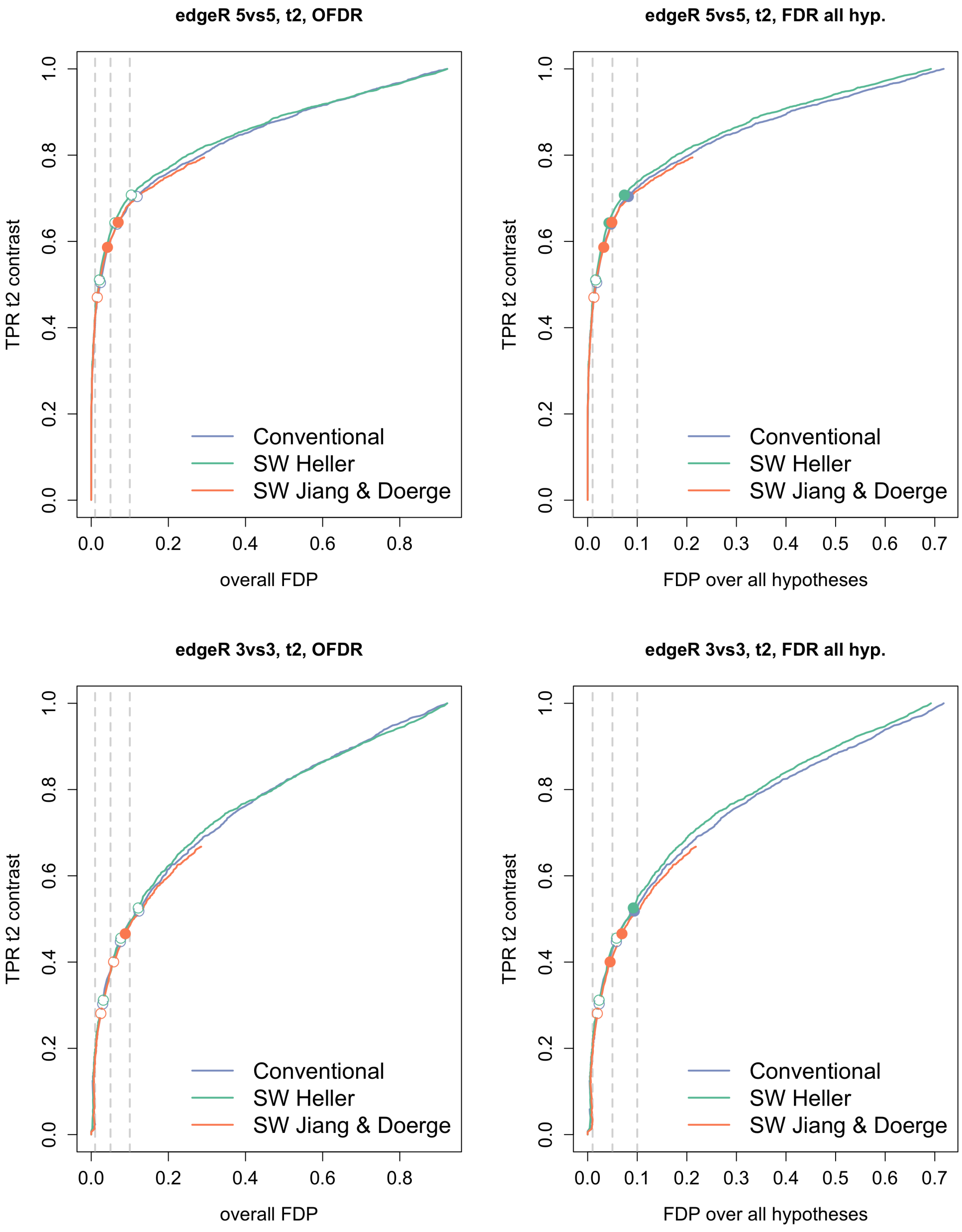

Figure S11: False discovery proportion - True positive rate (FDP-TPR) performance curves for the contrast at the second timepoint with the edgeR analysis. Top row: comparisons with five samples, bottom row: comparisons with three samples in every condition, left panels: performance of the overall FDR control and right panels: performance of FDR control across all hypotheses. The three points on the curves represent nominal FDR cut-offs at 1%, 5% and 10% and are filled if the empirical level is below its nominal level. A comparable sensitivity is observed across the three methods, however the Jiang method has conservative FDR control as suggested by the FDR working points.

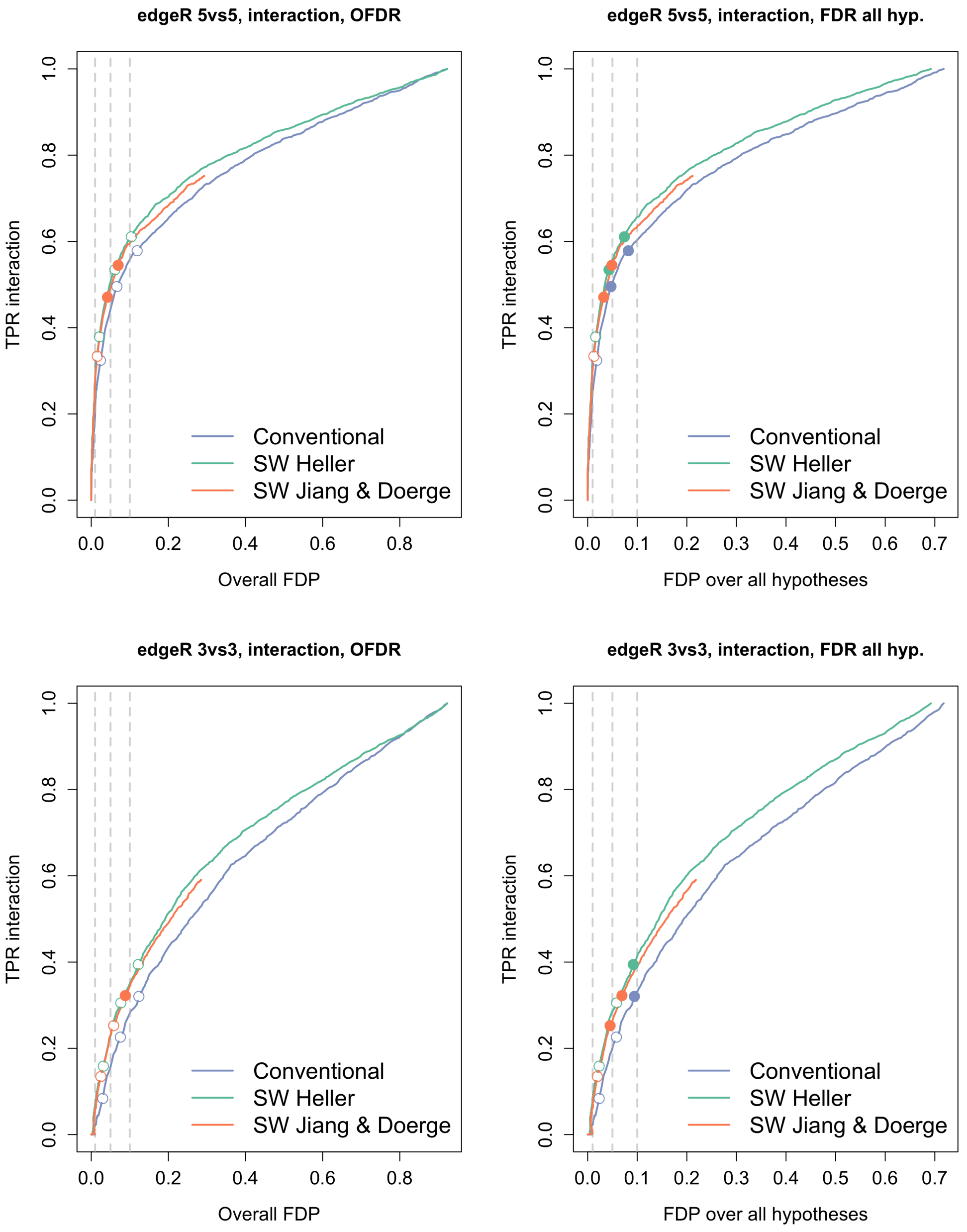

Figure S12: False discovery proportion - True positive rate (FDP-TPR) performance curves for the interaction effect in the edgeR analysis. top row: comparisons with five samples, bottom row: comparisons with three samples in every condition, left panels: performance of the overall FDR control and right panels: performance of FDR control across all hypotheses. The three points on the curves represent nominal FDR cut-offs at 1%, 5% and 10% and are filled if the empirical level is below its nominal level. A superior sensitivity is observed for the Heller method, outperforming the Jiang and conventional method. The Jiang method, however, provides a very conservative FDR control.

S-1.1.1 Case study

Table S1: Number of genes found in the Hammer dataset on a 5% FDR level with edgeR. The total number of analysed genes was 12893.

Procedure	t1	t2	Interaction	Stage I only	Unique genes
Standard	6869	6429	51	NA	7949
Stage-wise	6756	6425	761	117	7818

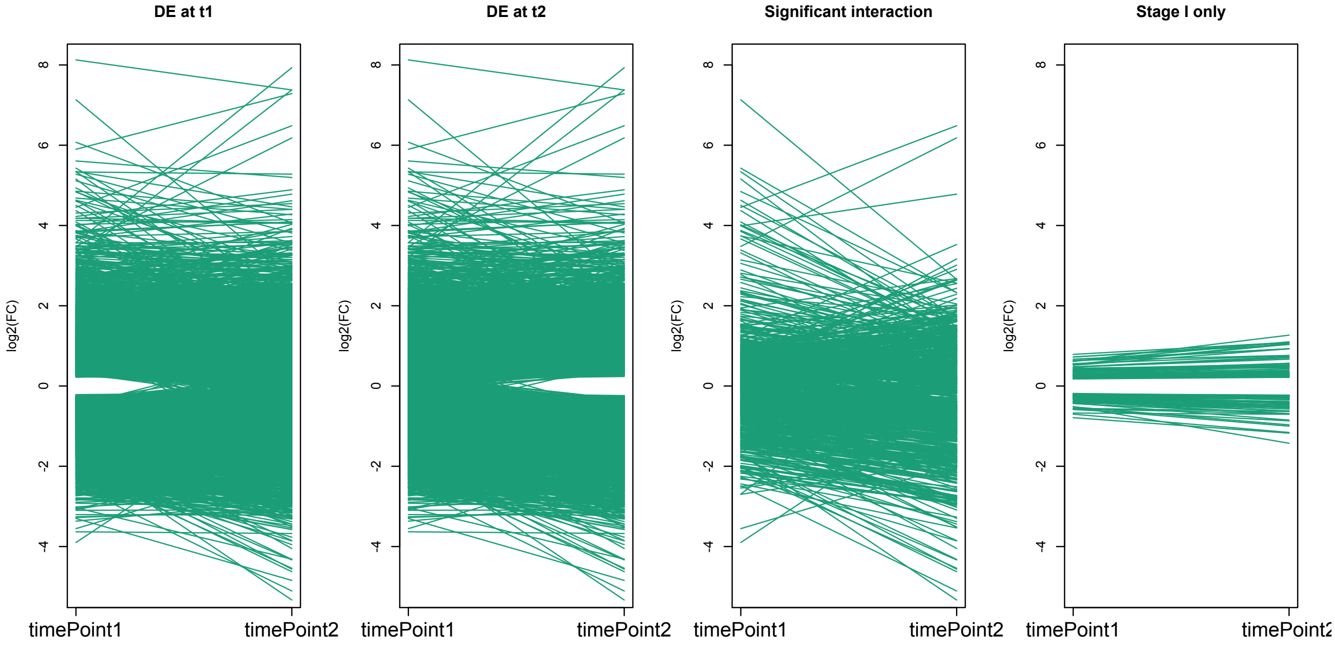

Figure S13: edgeR analysis of the DGE case study: genes significant in screening stage only have a constant, moderate fold change over time.

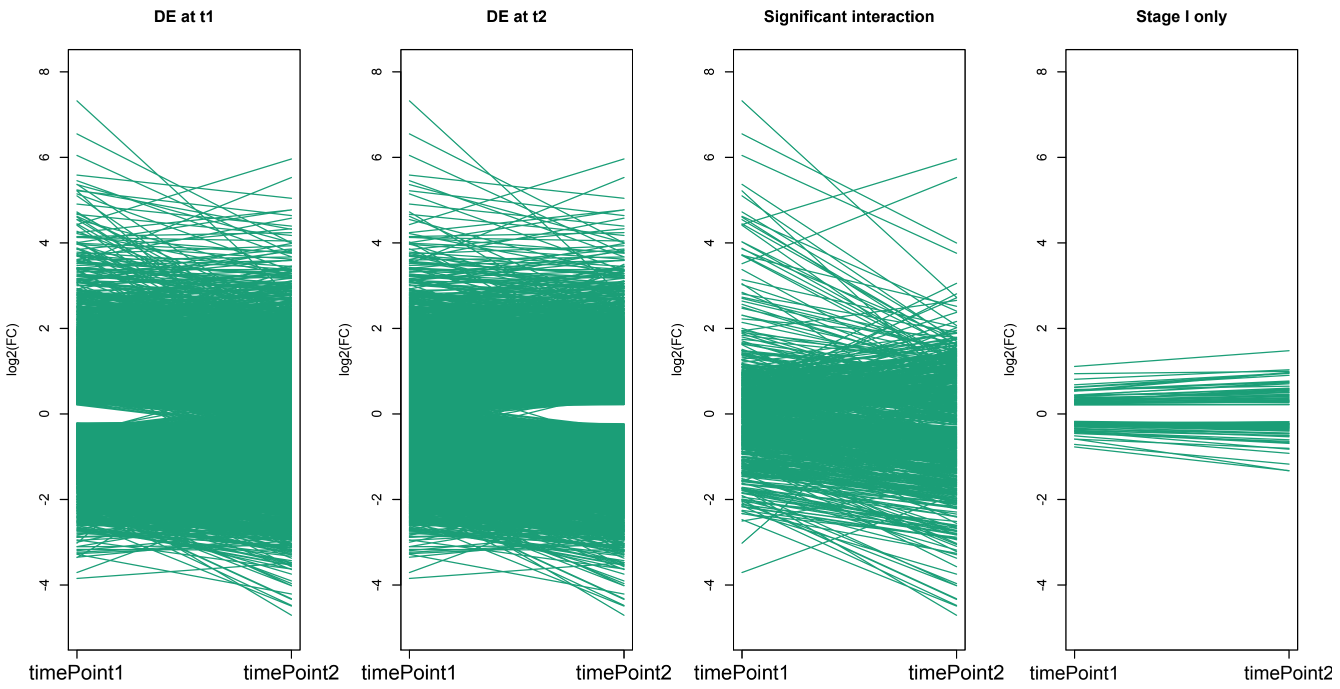

Figure S14: limma analysis of the DGE case study: genes significant in screening stage only have a constant, moderate fold change over time.

S-1.2 Differential transcript usage and differential transcript expression

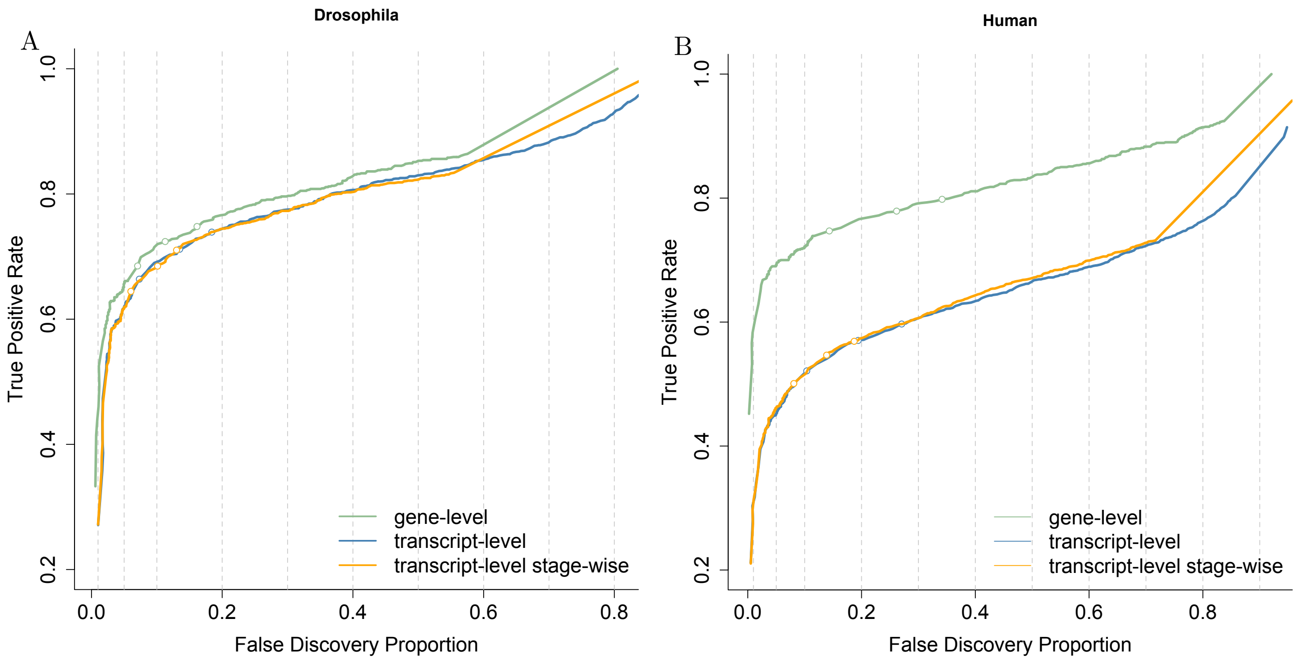

Figure S15: FDR-TPR performance curves for DTU analysis of the simulated data. Green curves represent the gene-level test and blue curves represent transcript-level tests. The orange curve represents the stage-wise transcript-level analysis. The three circles on the curves represent working points for a target FDR of 1%, 5% and 10%. (A) Performance curve for the Drosophila simulation shows an inflated FDR for both the conventional gene-level and transcript-level analysis. The stage-wise transcript-level analysis provides a better FDR control. (B) Performance curve for the human simulation shows an even worse FDR control on all levels. Similar to the Drosophila simulation, the transcript-level stage-wise analysis provides a better FDR control.

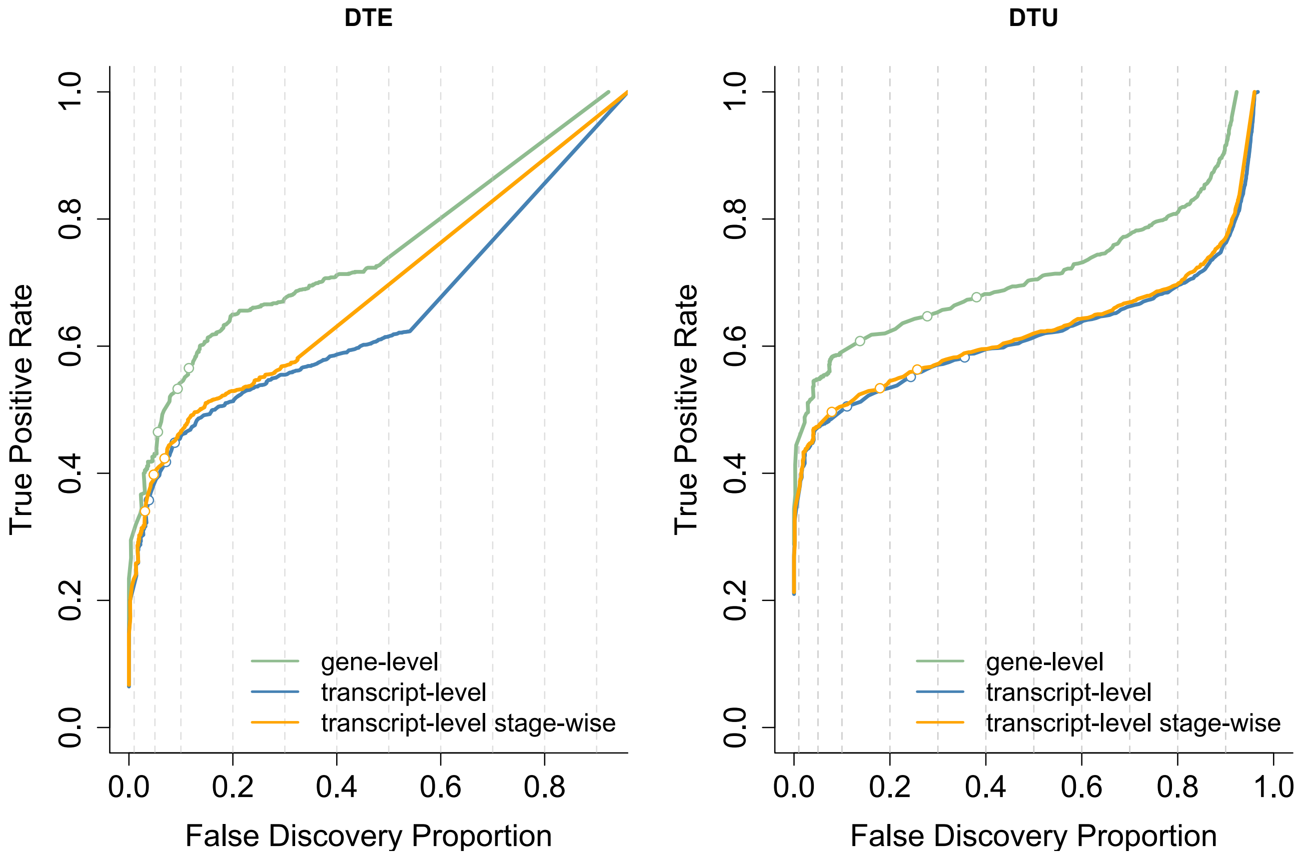

Figure S16: FDR-TPR performance curves for (A) DTE and (B) DTU analysis for the real data evaluation. The performance curves are similar to the curves obtained from the simulation study, suggesting good quality of the simulated data. Again, aggregated gene-level tests provide better sensitivity and the transcript-level stage-wise analysis has somewhat better performances compared to a regular transcript-level analysis in both settings. In general, FDR control seems to be harder in the DTU analysis.

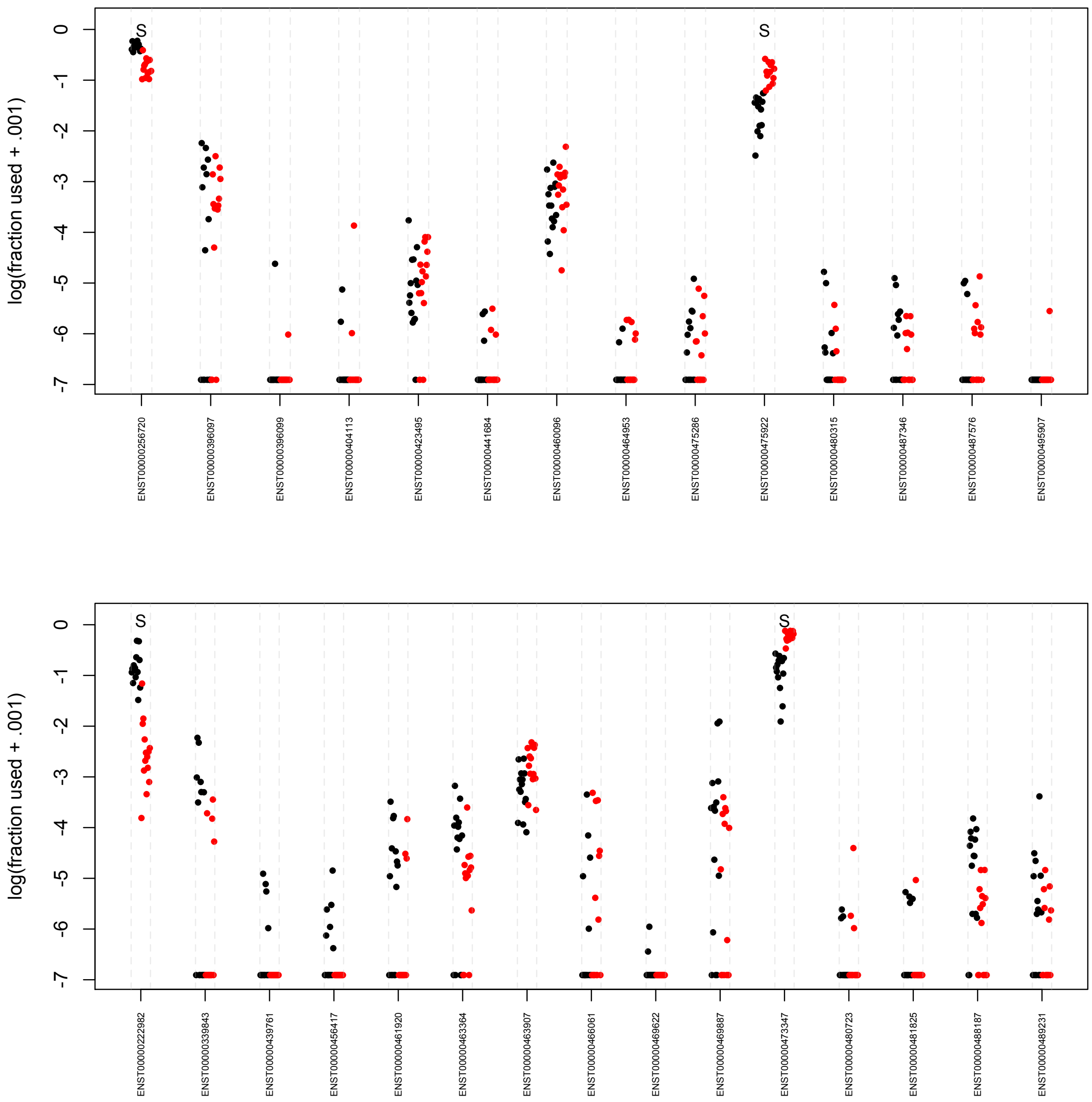

Figure S17: Transcript-level expression for all transcripts of the LPIN1 gene (top panel) and the CYP3A5 gene (bottom panel). The fraction used for every transcript is relative to the total expression of the genomic locus for a respective sample. Black symbols represent normal tissue and red symbols represent tumoral tissue. Significant differentially used transcripts according to the stage-wise analysis are indicated with an S at the top of the plot. For both genes a switch in isoform dominance is observed between normal and cancer cells.

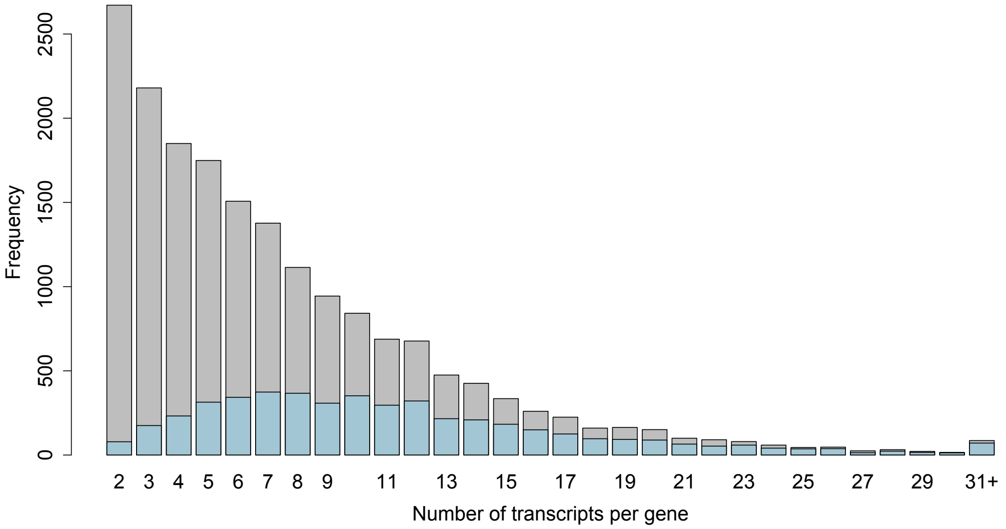

Figure S18: Distribution of number of transcripts per gene for all genes that have been analysed in the DTU prostate cancer case study dataset (grey). The blue portion of each bar represents the proportion of significant genes according to the stage-wise analysis. We observe that a higher number of transcripts for a gene increases its probability to be flagged as significant.

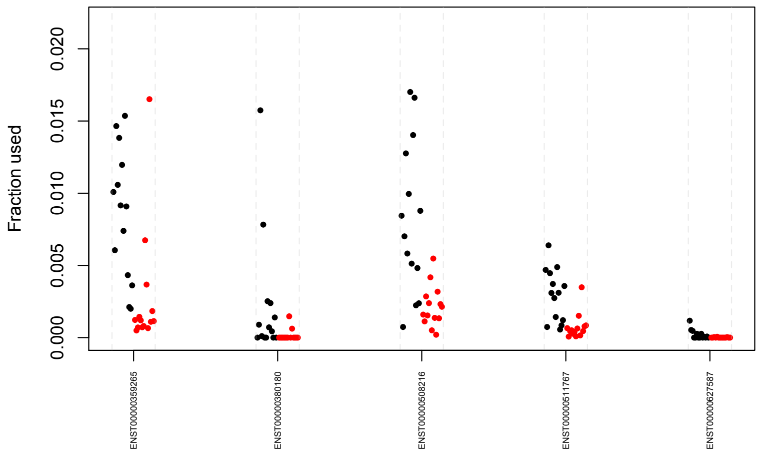

Figure S19: Transcript-level expression for significant lowly expressed transcripts of PDLIM5. The fraction used for every transcript is relative to the total expression of the genomic locus for a respective sample. Black points represent normal tissue and red points represent tumoral tissue. All transcripts shown are significantly downregulated as a compensation for the upregulation of the dominant transcript.

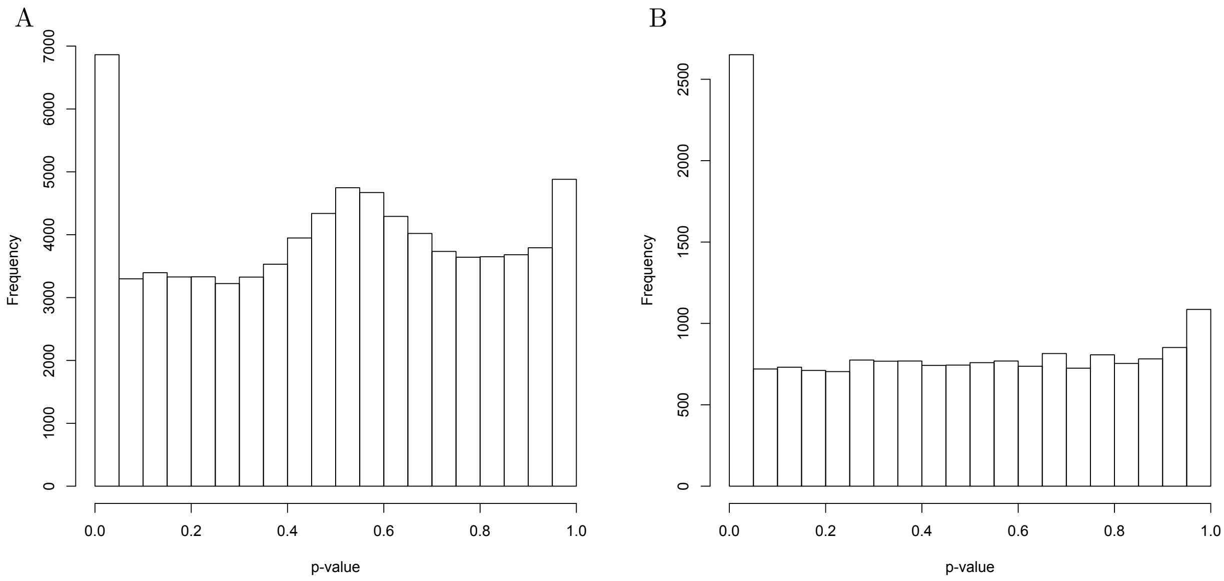

Figure S20: Transcript-level p-value distribution for the DEXSeq DTU analysis of the simulated data. (A) p-value distribution for the human data shows a non-uniform distribution for non-significant p-values including an overabundance of p-values in the 0.4 to 0.7 range. (B) p-value distribution for the Drosophila data shows a more uniform distribution for the non-significant p-values, suggesting more correct statistical inference.

References

[1] Jiang, H., Doerge, R.W.: A two-step multiple comparison procedure for a large number of tests and multiple treatments. Statistical applications in genetics and molecular biology **5**, 28 (2006). doi:10.2202/1544-6115.1223
